# Easy colorimetric detection of gadolinium ions based on gold nanoparticles: key role of phosphine-sulfonate ligands†

**DOI:** 10.1039/d0na00374c

**Published:** 2020-09-07

**Authors:** Marjorie Yon, Claire Pibourret, Jean-Daniel Marty, Diana Ciuculescu-Pradines

**Affiliations:** Laboratoire IMRCP, CNRS UMR 5623, Paul Sabatier University 118 route de Narbonne 31062 Toulouse France marty@chimie.ups-tlse.fr ciuculescu-pradines@chimie.ups-tlse.fr

## Abstract

The possibility to easily and rapidly assess the presence of Gd^3+^ ions in solution is of paramount importance in many domains like magnetic resonance imaging. In that context, the use of easy to implement colorimetric sensing probes based on gold nanoparticles (AuNPs) is of special interest. Herein, AuNPs functionalized with a commercial bis(*p*-sulfonatophenyl)phenyl phosphine ligand (BSPP) (AuNP@BSPP), bearing negatively charged sulfonate groups are used as a colorimetric sensing probe. The addition of Gd^3+^ ions onto these NPs was studied through UV-visible absorbance measurements, Quartz Crystal Microbalance with Dissipation monitoring (QCM-D) and transmission electron microscopy and compared with citrate covered AuNPs. We evidenced interactions between the Gd^3+^ ions and their water rich coordination sphere and sulfonate groups on the surface of AuNP@BSPP *via* electrostatic interactions and hydrogen bonding. These interactions induce the reversible aggregation of AuNP@BSPP in the presence of concentrations of Gd^3+^ ions at a μM level. We took advantage of this phenomenon to develop a simple and fast bench colorimetric assay for the detection of free Gd^3+^ ions, based on the determination of a flocculation parameter thanks to UV-visible measurements. Limits of detection and quantification were found equal to 0.74 μM and 4.76 μM of Gd^3+^ ions, respectively, with a high sensitivity that competes with conventional methods used for lanthanide detection.

## Abstract 2

Specific interactions between sulfonate groups of phosphine-sulfonate ligands on the surface of Au nanoparticles and Gd^3+^ ions allow the colorimetric detection of Gd^3+^ ions at the μM level.

## Graphical abstract

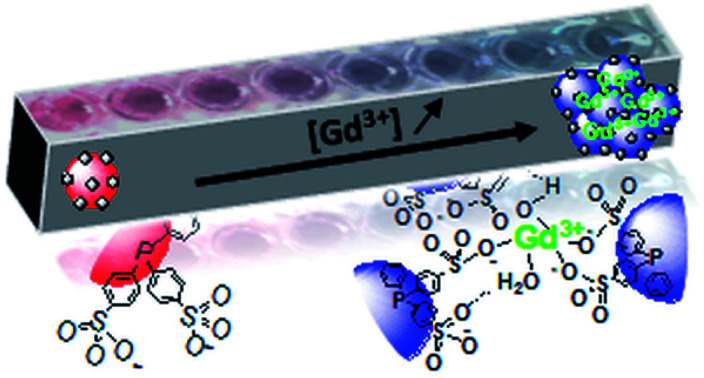

## Introduction

Lanthanide ions have seen their field of application increased in recent years, for their catalytic, optical and magnetic properties. Hence most of the clinically used contrast agents in magnetic resonance imaging are based on the use of gadolinium molecular complexes like Dotarem®, Magnevist®,*etc*. Nevertheless, developing new gadolinium-based contrast agents with enhanced relaxivity properties while avoiding release of toxic free gadolinium ions in human tissue is still of paramount importance.^1^ The possibility to easily and rapidly assess the presence of such free gadolinium ions in the contrast agents requires the development of efficient analytical methods and strategies to detect gadolinium. Numerous methods were used, including UV-visible absorbance and fluorescence spectroscopy, electrospray ionization mass spectrometry (ESI-MS) and inductively coupled plasma mass spectrometry (ICP-MS).^2^ These methods, although efficient, are often difficult to implement and do not enable for rapid routine determinations.

In that context the use of gold nanoparticles (AuNPs) as colorimetric sensing probes have piqued much interest^3^ in addition to their application in biomedical imaging.^4^ AuNPs are still today one of the largest studied inorganic colloids, due to their interesting photo-physical properties, easy preparation and functionalization.^5^ The ability to detect, selectively and quantitatively, metal ions relies on the interactions of metal ions with these AuNPs that modify the dielectric properties of the ligand shell and eventually induce aggregation. Both phenomena responsible for the change of optical properties of colloidal solutions, can be further related to the concentration of ions in solution. The affinity and selectivity towards metal ions require to have access to AuNPs with both a well-controlled morphology (size and shape) and the suitable nature of the ligands present at their surface. However, available synthetic methods are still unsuited to have direct access to such NPs due to three main limitations: (i) the control of NP core size and shape often requires stringent growth conditions relying on the use of specific solvents, and reducing and functionalizing agents, (ii) a purification step is needed between the synthesis of the core and the surface modification, and (iii) the surface modification by ligand exchange or chemical reaction is often limited and is time-consuming requiring special design of the ligands and precise control of the functionalization process involving frequently inadvertent aggregation of the particles.

**Fig. 1:**
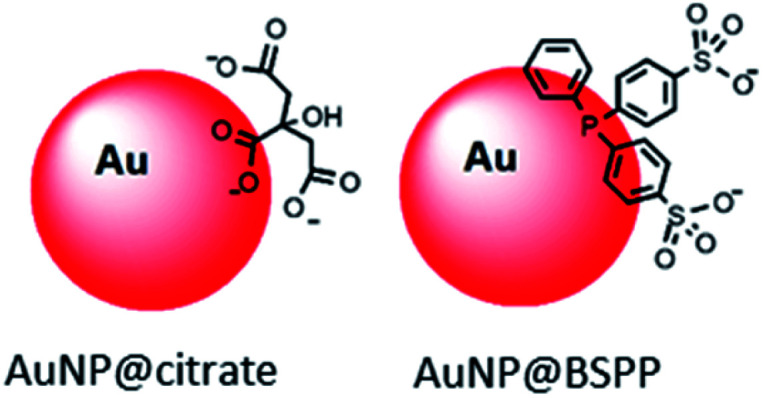
Schematic representation of AuNP@citrate and AuNP@BSPP described in this article.

Here in order to have access to AuNPs with a controlled morphology and functionalization to be used as colorimetric probe, we decided to start from 20 nm AuNPs synthesized by the Turkevich and Frens method,^6,7^ denoted as AuNP@citrate. Despite the convenience and reproducibility of this synthesis, the resulting colloidal solution displays poor stability due to the weak binding of the citrate ions to the gold surface. Bis(*p*-sulfonatophenyl)phenylphosphine (BSPP) is added to lead to more stable negatively charged modified AuNPs,^8^ denoted as AuNP@BSPP (Fig. 1).

Despite the large use of AuNP@BSPP, there is no study concerning their behavior in the presence of lanthanide ions, so far. We demonstrate in this article the high sensitivity of the optical properties of these NPs to the addition of Gd^3+^ ions. UV-visible spectroscopy and quartz crystal microbalance with dissipation monitoring (QCM-D) measurements enable the understanding of the mechanism responsible for this phenomenon. Finally, we took advantage of the behavior of AuNP@BSPP to validate a highly sensitive colorimetric test for the detection of gadolinium ions. We illustrate this method to quantify free Gd^3+^ ions present in the colloidal systems obtained from polymers and Gd^3+^ ions, and compare the results obtained with the ones issued from ICP-MS analysis.

## Results and discussion

### Synthesis and characterization of gold nanoparticles

Citrate stabilized gold nanoparticles (AuNP@citrate) were prepared through the reduction of HAuCl_4_ by citrate ions using the Turkevich method.^5^ The citrate ligand was then replaced by a water-soluble phosphine ligand bearing sulfonate anionic groups, BSPP, which binds to the surface of AuNPs *via* phosphorus' lone electron pair (see details in the Experimental section).^9–11^ The interaction between the phosphorus atom and the AuNP surface and, in addition, steric hindrance of the bulky aromatic rings of BSPP enable to obtain gold nanoparticles (AuNP@BSPP) with enhanced colloidal stabilization properties.^8^ AuNP@BSPP were thus exploited by Alivisatos^12^ to ensure efficient functionalization of AuNPs with ssDNA. AuNP@BSPP remain stable even for high nanoparticle concentrations (up to 100 times more concentrated than the AuNP@citrate) and maintain their red wine color. Another advantage of using BSPP as a capping agent is the ability to easily redisperse AuNP@BSPP precipitated by the addition of salt or by centrifugation.^9^

**Fig. 2:**
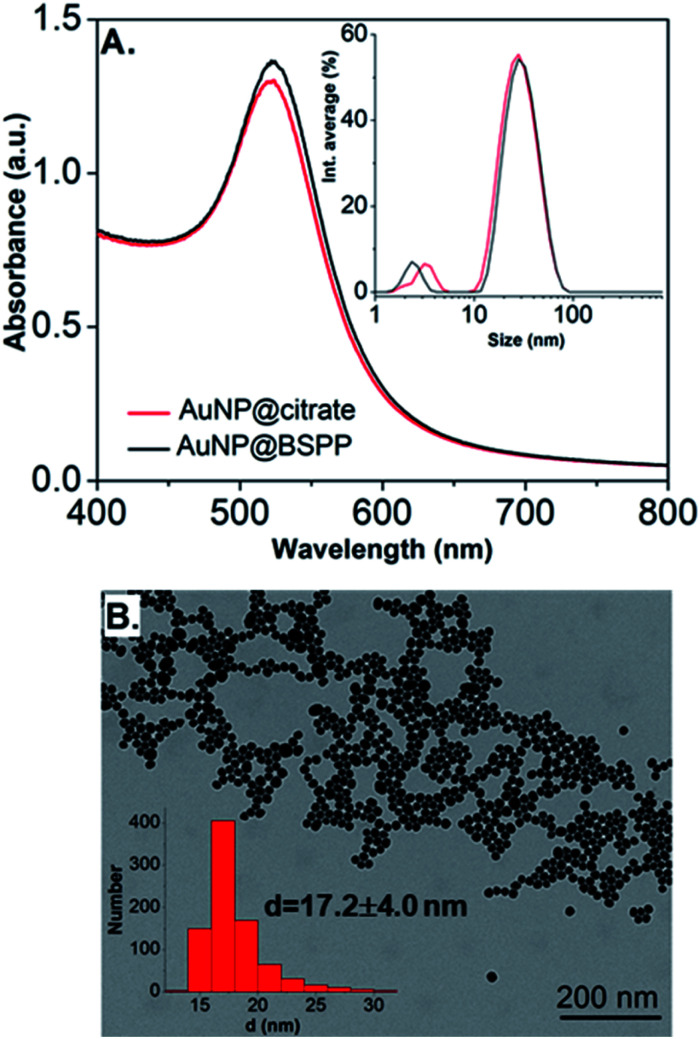
(A) UV-visible spectra and intensity-averaged hydrodynamic diameter distribution (inset) estimated from dynamic light scattering measurements of AuNP@citrate (red curve) and AuNP@BSPP (black curve). A small peak is observed in DLS at sizes lower than 5 nm due to rotational diffusion of particles.^14^ (B) Transmission electron microscopy image of AuNP@BSPP and the respective size distribution histogram (inset).

UV-visible absorption spectra of AuNP@citrate and AuNP@BSPP solutions are shown in Fig. 2A. In the case of AuNP@citrate, a surface plasmon resonance band is clearly visible with a maximum absorbance at 522 nm.^5^ An estimation of the diameter of the AuNPs can be obtained from the ratio of the absorbance measured at the maximum of the peak to the one at 450 nm as detailed in the supporting information (ESI Fig. S1†).^12^ From this, an average diameter of 17 nm could be estimated in agreement with the size found by transmission electron microscopy (TEM) (ESI Fig. S2†). A slight increase of the maximum absorbance of the surface plasmon resonance peak and a red shift from 522 to 523 nm is observed upon addition of the phosphine ligand due to changes of the dielectric environment around nanoparticles.^5,13^ An average diameter of 17.2 ± 4.0 nm was measured from TEM images (Fig. 2B) in good agreement with pristine AuNP@citrate. In addition, the efficiency of ligand exchange was assessed from dynamic light scattering (DLS) and by sedimentation field-flow fractionation (SdFFF) measurements (see Fig. S3 and S4 in ESI†). An intensity-averaged hydrodynamic diameter equal to 31 ± 11 nm and 33 ± 11 nm was obtained for the AuNP@citrate and AuNP@BSPP, respectively (inset in Fig. 2A). The growth or aggregation of the NPs during the ligand exchange process can therefore be excluded. Zeta potentials of AuNP@citrate and AuNP@BSPP solutions are −35 ± 3 mV and −40 ± 5 mV, respectively, evidencing, as expected, a negative surface charge of the NPs (see ESI Fig. S5†).

### Addition of gadolinium ions on gold nanoparticles

**Fig. 3:**
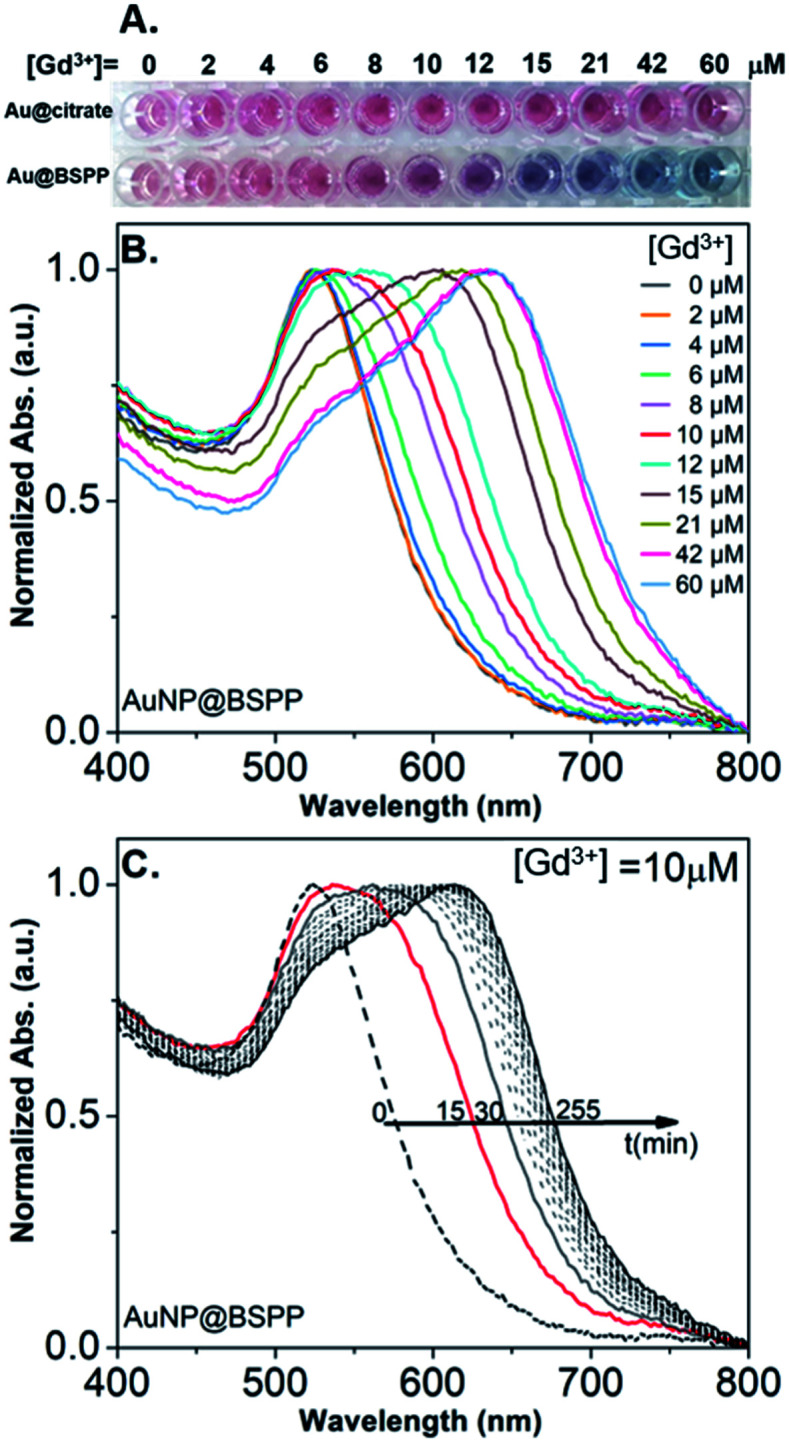
(A) Photographs showing the color response of AuNP@citrate (first line) and of AuNP@BSPP (second line) solutions after incubation with increasing concentrations of Gd(NO_3_)_3_. Both AuNP solutions were used at the same concentration of 0.14 mM gold ions. (B) Evolution of UV-visible spectra of AuNP@BSPP in the presence of increasing concentrations of Gd(NO_3_)_3_ at *t* = 15 min. (C) Temporal evolution of UV-visible spectra of AuNP@BSPP for a concentration of Gd^3+^ ions equal to 10 μM. Spectra of AuNP@citrate are given in ESI, Fig. S8.†

In order to assess the possibility to use AuNP@BSPP as a colorimetric sensing probe for Gd^3+^ ions, we have first evaluated the effect of adding Gd(NO_3_)_3_ solutions of increasing concentrations. As depicted in Fig. 3A, the addition of Gd(NO_3_)_3_, from 2 to 60 μM, on AuNP@BSPP induced a progressive color change from red to blue, higher concentrations inducing spontaneous precipitation. However, no color change was observed for the same range of Gd(NO_3_)_3_ concentrations added to AuNP@citrate. For these AuNP@citrate, a color change required the addition of a higher concentration of Gd(NO_3_)_3_ above 100 μM (see ESI Fig. S6†).

Therefore, AuNP@BSPP have a sensing limit for Gd^3+^ ions significantly lower than AuNP@citrate. Besides, it questions the mechanism of interaction of Gd^3+^ ions with the surface of AuNPs leading to the aggregation of AuNP@BSPP.

**Fig. 4:**
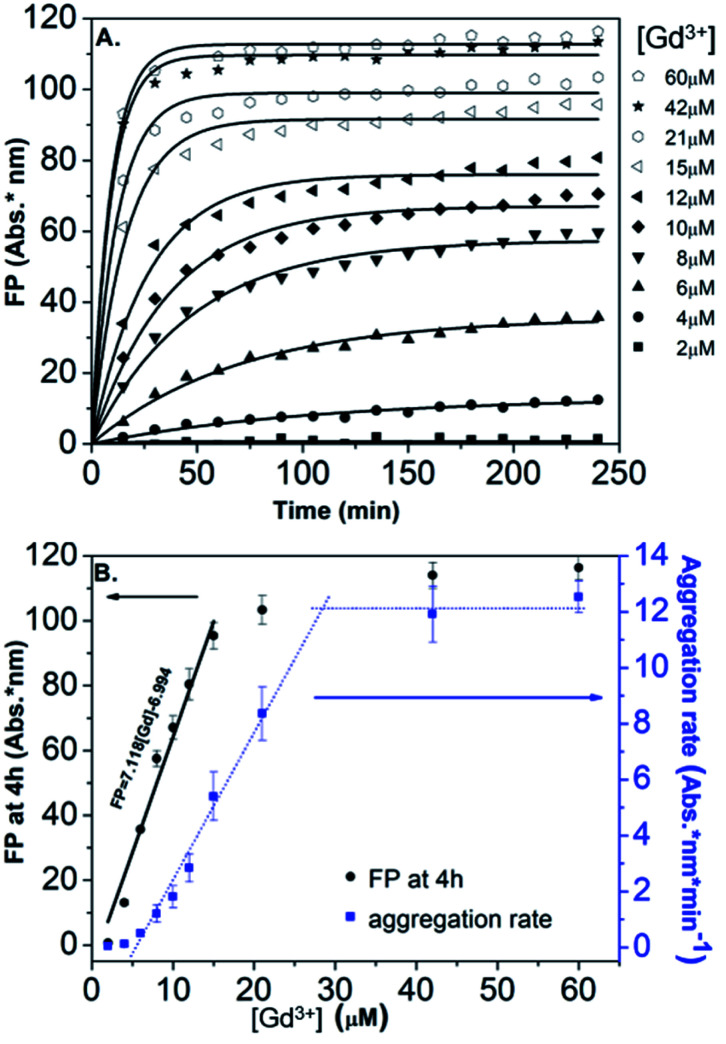
(A) Time dependence of the FP of AuNP@BSPP plotted for increasing concentrations in Gd(NO_3_)_3_ together with the best fit to eqn (1). (B) Corresponding FP at 4 h (black dots) and aggregation rate (blue dots) plotted *vs.* concentrations of Gd(NO_3_)_3_.

In order to obtain further information on the mechanism behind the observed changes, the study of the evolution of the absorbance spectra was carried out at different concentrations of Gd(NO_3_)_3_. In agreement with the visual observation, the wavelength corresponding to the maximum of the peak is progressively red-shifted from 523 nm up to 636 nm for concentration of Gd(NO_3_)_3_ increasing from 0 to 60 μM (Fig. 3B and ESI Fig. S7†). Additionally, the recording of the temporal evolution of the absorbance spectra every 15 minutes during 4 hours shows an evolution of the global aspect of the spectra (Fig. 3C). In contrast to AuNP@BSPP, whatever the concentration of Gd(NO_3_)_3_ and regardless of the measurement time, no evolution is observed in the case of AuNP@citrate solutions (see ESI Fig. S8†). In order to analyze quantitively these results, we further used the semiempirical spectroscopic flocculation parameter (FP) as described by Mayya *et al.*^15^ FP was calculated from normalized spectra by calculating the integration between 600 and 800 nm from which the integrated absorption for pristine AuNP@BSPP between the same limits was subtracted (for more details see ESI Fig. S9†). Fig. 4A illustrates the time dependence of the flocculation parameter of AuNP@BSPP at different concentrations in Gd(NO_3_)_3_. AuNP@BSPP aggregation increases with time to reach a plateau and at increasing rates with the Gd(NO_3_)_3_ concentration. The experimental kinetic data were analyzed by fitting the flocculation parameter by using a mono-exponential eqn (1)

1 FP = FP_max_(1 − exp(−*t*/*τ*_F_))

where FP_max_ represents the plateau value and *τ*_F_ is a characteristic time describing the aggregation kinetics.^16^

Fig. 4B illustrates the evolution of the flocculation parameter recorded at different concentrations for *t* = 4 h. First, a continuous increase is observed when the concentration of Gd(NO_3_)_3_ is varied from 2 to 18 μM. These results evidence the high detection capacity of AuNP@BSPP for considerably low Gd(NO_3_)_3_ concentrations and could be easily exploited to develop a colorimetric test. The validation of this approach to determine gadolinium content was carried out following the recommendations of standard ISO 5725 and 17025. For the chosen concentration of NPs, the method presents a linear range: 0 to 18 μM (FP = 7.118 × [Gd] − 6.994; *R*^2^ = 0.974). The detection limits are [Gd]_LD_ = 0.74 μM and FP_LD_ = 5.27 nm and the quantification limits are [Gd]_LQ_ = 4.76 μM and FP_LQ_ = 33.90 nm. The repeatability of the method was estimated from a series of nine independent measurements. From this, the coefficient of variation of repeatability, *i.e.* the ratio of the standard deviation to the mean, was calculated and is equal to 8.8%. This gives a measure of the dispersion of data points around the mean (see details of the calculation in the ESI†). This calibration line will be used in the last part of this article.

Moreover, in order to follow the effect of the concentration of Gd(NO_3_)_3_ on the relative rate of AuNP@BSPP aggregation, the maximum slope (*F*_max_/*τ*_F_) of the flocculation curves was also plotted *vs.* Gd(NO_3_)_3_ concentration^17^ (Fig. 4B). At low concentrations, the aggregation rate is small and increases progressively when the concentration is varied from 6 to 28 μM. Beyond this concentration, the aggregation rate reaches a plateau and becomes constant. The observed aggregation of AuNP@BSPP may reflect the well-known process of coagulation induced by the addition of electrolytes. This process, described by the DLVO theory, predicts, as observed here, a slow and fast aggregation regime. The threshold salt concentration needed to induce rapid aggregation (here 28 μM) is referred to as the critical coagulation concentration (CCC).^18^ The Schulze–Hardy rule demonstrates a relationship between CCC of colloids and the valence of extra counter ionic electrolytes (z).^19^ Accordingly, divalent and trivalent ions are much more capable of causing precipitation of colloids from their suspensions than the monovalent ones.

**Fig. 5:**
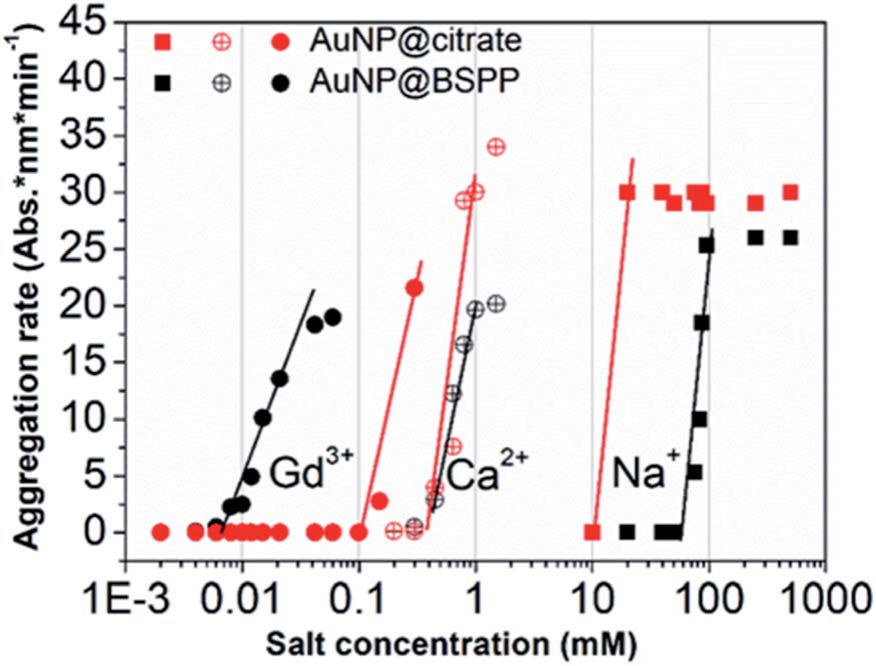
Comparison of aggregation behavior of AuNP@BSPP (black plot) with that of AuNP@citrate (red plots) in the presence of mono, di and trivalent cations (NaCl, Ca(NO_3_)_2_ and Gd(NO_3_)_3_) respectively.

The influence of the valence of cations on the relative aggregation rate was further studied in order to gain more insights into the aggregation process of AuNP@BSPP, in comparison to that of AuNP@citrate. In addition to Gd^3+^ ions, we studied the influence of Ca^2+^ divalent and Na^+^ monovalent ions. The effect of the concentration of these different cations on the relative aggregation rate is depicted in Fig. 5 and ESI Fig. S10.†

For the three ions and for both types of NPs, the characteristic slow and fast aggregation regimes are evidenced. Moreover, the values of CCCs lie between 10 and 100 mM for Na^+^ ions, around 1 mM for Ca^2+^ ions and between 0.01 and 0.1 mM for Gd^3+^ ions. The CCCs which separate the two regimes decrease in order of monovalent, divalent and trivalent ions which is in agreement with the Schulze–Hardy rule.^18^ For the monovalent Na^+^ ions the CCC for AuNP@BSPP is higher than the value for AuNP@citrate: 100 mM and 50 mM respectively. This difference reflects the higher negative charge (zeta potential −40 ± 5 mV) and the higher stability of AuNP@BSPP compared with the AuNP@citrate (zeta potential −35 ± 3 mV). This is in agreement with an aggregation mainly induced by the compression of the electrical double layer of the NPs. For the divalent ions the same CCCs are observed for the two kinds of NPs, indicating a stronger adsorption of divalent ions to the surface than the monovalent ones.^18^ Noteworthily, this tendency is reversed for Gd^3+^ ions. The CCC of AuNP@BSPP of 0.03 mM is one order of magnitude lower than for the AuNP@citrate (0.3 mM). This reversed trend could be attributed to a specific and more effective interaction of the Gd^3+^ ions with the negative sulfo-groups stabilizing the AuNP@BSPP than with the citrate groups stabilizing the AuNP@citrate.^20^

### Further insights into Gd^3+^ ions adsorption through quartz crystal microbalance characterization

Both the large size of AuNPs leading to low molecular tumbling of the ligands together with the paramagnetic character of Gd^3+^ ions do not allow an easy study of these systems by NMR spectroscopy.^21^ In order to obtain more details on the interaction phenomena between AuNPs and Gd^3+^ ions, complementary studies using a quartz crystal microbalance with dissipation monitoring (QCM-D) were performed. QCM-D is a powerful analytical strategy for characterizing the adsorption of molecules on 2D surfaces^22^ and it was already successfully used to study colloidal nanoparticle solutions.^23^ With a possibility to detect mass changes as low as 1 ng cm^−2^, QCM-D was especially used to probe the interaction with macromolecules, such as proteins, nucleic acids, lipid bilayers, bacteria, and polymers.^23–28^ The studies reporting the adsorption of low molecular weight molecules are more scarce because of measurement difficulties related to low grafting density or limited binding affinities.^29^ However, the adsorption of fluorescein-5-isothiocyanate onto gold surfaces passivated either with citrate or with BSPP^30^ and the detection of Cu^2+^ ions^31^ were recently studied through QCM-D sensing experiments.

The interaction of citrate and BSPP with bare gold surfaces was studied first. Solutions of citrate (24 mM in water, pH 5.5) and BSPP (24 mM in water, pH = 6.5) were deposited at a flow rate of 40 μL min^−1^. A decrease in frequency was observed suggesting the attachment of both molecules on the gold surface (ESI Fig. S11.1†). A surface coverage of 0.3 molecules per nm^2^ was calculated using the Sauerbrey equation for both citrate and BSPP. However, during the rinsing step almost all the adsorbed citrate was washed out, causing the frequency to increase progressively until almost reaching its initial value. This is consistent with a weak interaction of citrate with the gold surface *via* Au–COO^−^ electrostatic interactions estimated to be 2 kcal mol^−1^.^32,33^ In contrast, after the washing step, half of the BSPP molecules remain on the gold surface leading to an apparent surface coverage of retained phosphine equal to *Γ* = 0.14 molecules per nm^2^. This difference with citrate is expected according to the stronger Au–P interaction which is about 15–20 kcal mol^−1^.^34^ Additionally, BSPP bearing two Ph–SO_3_^−^ functions (p*K*_a_ of −2.8), suggests cooperative or competitive participation of Au–SO_3_^−^ electrostatic interactions during the adsorption process. This could induce steric hindrances on the surface that could be responsible for low adsorption behavior and partial desorption of BSPP as reported for QCM-D studies.^35,36^

**Fig. 6:**
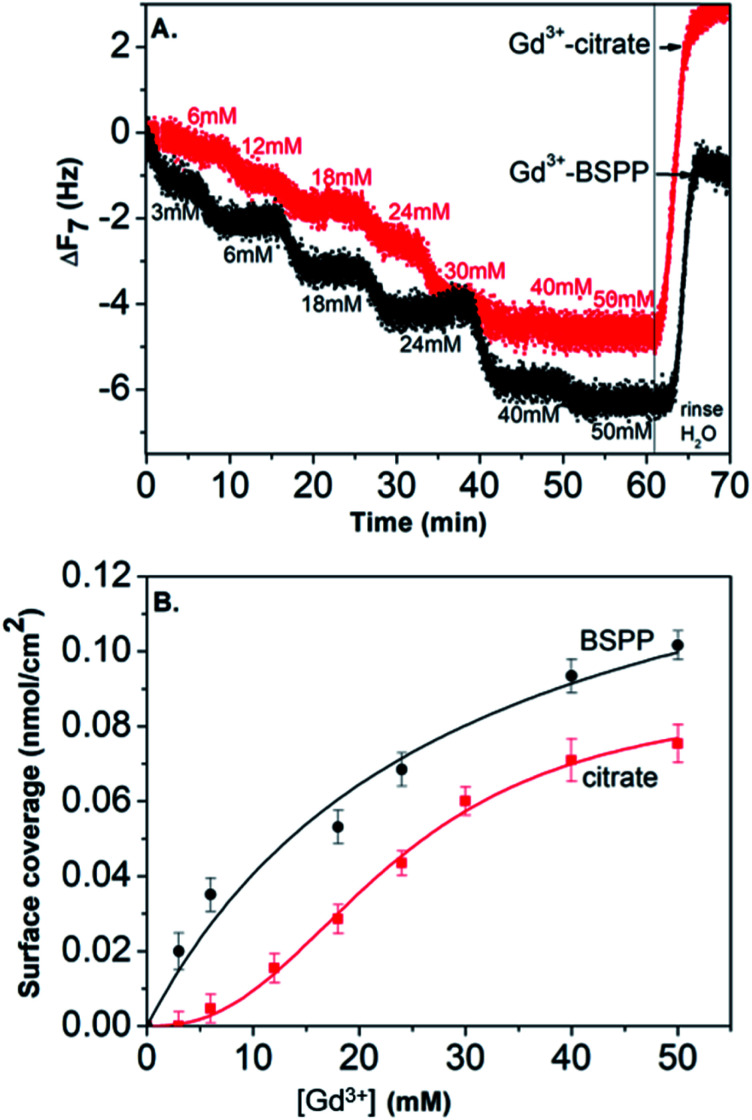
(A) Frequency shift with time (Δ*F*_7_ 7^th^ overtone) for Gd^3+^ ion adsorption. The measurements were carried out as sequential deposition of increasing concentration of Gd^3+^ ions. (B) Plot of the surface coverage *versus* concentration of Gd^3+^ ions, fitted using the Langmuir–Freundlich model (for the citrate precoated surface) and Langmuir model (for the BSPP precoated substrate).

In view of these results, the preparation of gold substrates for the subsequent Gd^3+^ ion deposition consisted first in their overnight incubation with citrate and BSPP solutions (24 mM in water). Concerning the further adsorption of Gd^3+^ ions, in order to avoid desorption of citrate ions, injection of Gd^3+^ ions was performed by keeping constant the concentration of citrate (24 mM) throughout the experiment.^23^ In the case of BSPP, the substrate was rinsed, equilibrated before the adsorption of Gd^3+^ ions and eluted with water. Fig. 6A and ESI Fig. S11.2† show the evolution of Δ*F* as a function of time when the measurements were carried out as sequential depositions of solutions of Gd(NO_3_)_3_ of increasing concentrations. The resulting surface coverage, calculated with the Sauerbrey equation, as a function of Gd^3+^ concentration is depicted in Fig. 6B. These curves were subsequently fitted using Langmuir and Langmuir–Freundlich isotherm models describing the interaction of the adsorbing molecule, *i.e.* the Gd^3+^ ion, with the adsorption surface sites (see ESI Fig. S11.3† for details).^37,38^

For the adsorption onto the BSPP coated substrate the experimental data were best fitted by the Langmuir isotherm model (*R*^2^ = 0.93) that assumes gradual saturation of sorption sites, without lateral interactions between adsorbed molecules, producing monolayer adsorption on a homogeneous adsorbent surface. This suggests a strong specific interaction of Gd^3+^ ions with the phosphine coated substrate. Rinsing with pure water increases the frequency to the level registered for the phosphine-coated substrate (see Fig. 6A) thus confirming that the phosphine was not washed out during the experiment. Additionally, the results of the experiments conducted on the adsorption of Gd^3+^ on a bare gold substrate (see ESI Fig. S11.4†) show differences, excluding then a potential displacement of the phosphine passivating layer during the Gd^3+^ adsorption. In the case of the interaction of Gd^3+^ ions with a citrate coated substrate, the adsorption of the Gd^3+^ ions onto the citrate coated substrate did not obey a Langmuir type isotherm model. The fitting parameter *n* = 2.4 of the Langmuir–Freundlich isotherm (*R*^2^ of 0.998, see Table S11.3†) may be identified as describing a cooperative interaction.^39^ This probably accounts for a mechanism of adsorption *via* the complexation between the Gd^3+^ ions and the carboxylic groups of the citrate ligand.^40^

**Fig. 7:**
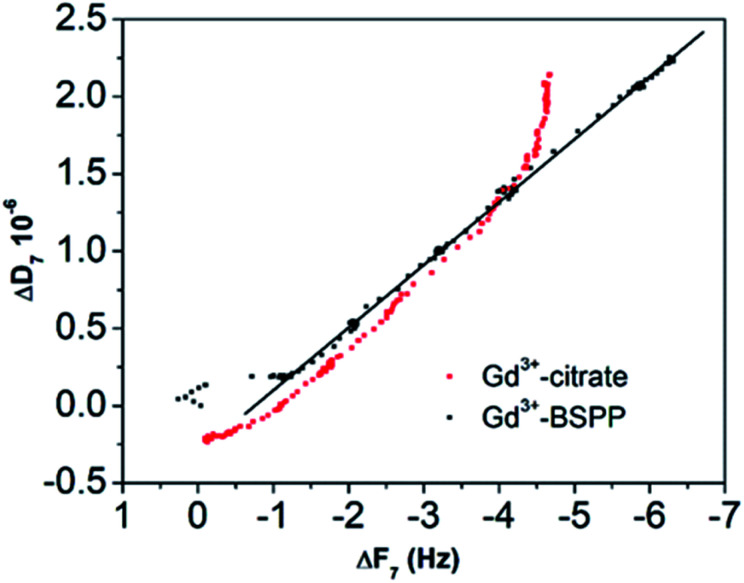
Δ*D versus* Δ*F* plot for the Gd^3+^ ions adsorbed onto the citrate (red dots) and onto the BSPP coated gold surfaces (black dots), respectively.

This result stresses that the adsorption mechanisms onto the two surfaces are significantly different and confirms the observations on the NPs. Different adsorption scenarios on the two different surfaces are also confirmed by the plot of the measured energy dissipation per unit of mass (Δ*D*) *vs.* frequency (Δ*F*)^41,42^ (Fig. 7).

**Fig. 8:**
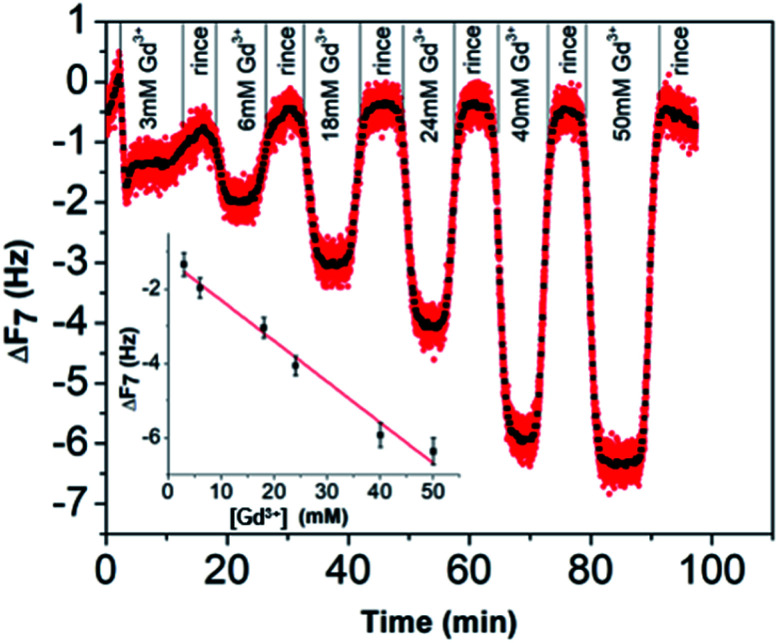
Response of the QCM-D gold sensor pre-coated with the BSPP molecules to the increasing concentration of Gd^3+^ ions. A washing step with water between each Gd^3+^ ion deposition step was performed.

Concerning the BSPP coated surface, the Δ*D*/Δ*F* ratio remains constant suggesting an interaction of Gd^3+^ ions as a single layer *via* specific interactions.^42^ On the contrary, for citrate coated surfaces, the nonlinear variation of the Δ*D* with the Δ*F* can be related to the formation of loosely bound conformations between citrate or/and its complexes with the Gd^3+^ ions and the gold substrate.^42^ These weak interactions with the gold substrate induce a full desorption of citrate and Gd^3+^ ions after rinsing the surface with pure water (see Fig. 6A and ESI Fig. S11.5†). As a consequence of the specific interaction between Gd^3+^ ions and the phosphine coated substrate, measurements as sequential depositions of increasing Gd^3+^ concentrations with a washing step in between each deposition step show full reversibility, and short responses and recovery times (Fig. 8). Furthermore, the frequency response *versus* Gd^3+^ ion concentration shows a satisfactory linear behavior in-between the experimental concentration range (Fig. 8 inset).This QCM-D experiment accounts for the sensing performance of the Gd^3+^–Au–BSPP 2D-system and demonstrates the relevance to address an easier method to implement a bench test, based on the Gd^3+^–AuNP@BSPP 3D system.

### Origin of aggregation of AuNP@BSPP by Gd^3+^

Pristine AuNP@BSPP solutions showed a *ζ* potential value equal to −40 ± 5 mV. After addition of 25 μM of Gd^3+^ ions, zeta potential of Gd^3+^–AuNP@BSPP decreased up to −8 ± 6 mV. As only a small decrease of pH was measured (from 7 to 6.4), this reduction can be mainly explained by the NP surface charge screened by Gd^3+^ ions.^43^ This *ζ* potential change might be responsible for their aggregation and color change. In addition, the results presented in previous sections demonstrate that a strong specific interaction between Gd^3+^ ions and the negatively charged BSPP ligands takes place. The hard nature of the sulfonate groups favours the interaction with the hard rare-earth Gd^3+^ ions, although sulfonate groups are regarded as weak ligands. However, the weak coordination nature of sulfonate groups makes their coordination mode very sensitive to the chemical environment.^44,45^ The aggregation of the AuNP@BSPP is made possible by creating interactions with the sulfonate groups through cooperative electrostatic interactions and hydrogen bonding. These interactions are described in the literature as labile interactions that do not require replacement of the ligands like water or hydroxy groups (from the partial hydrolysis of Gd^3+^ ions at pH > 5.7) present in the first coordination sphere of the Gd^3+^ ion.^46^ Numerous examples of this behaviour can be found in the crystal structures of lanthanide ion complexes of sulfonatocalixarenes, leading to the identification of multiple coordination spheres around metal ions.^44,45^ The interaction between Gd^3+^ ions and an increased number of styrene sulfonate groups accounted for the strong polystyrenesulfone–Gd^3+^ binding.^47^ Here, hydrogen bonds are formed with the ligands from the first coordination sphere of Gd^3+^ ions. These interactions can thus be easily broken by changing the chemical environment as demonstrated by QCM-D experiments during the rinsing step (*vide supra*).

**Fig. 9:**
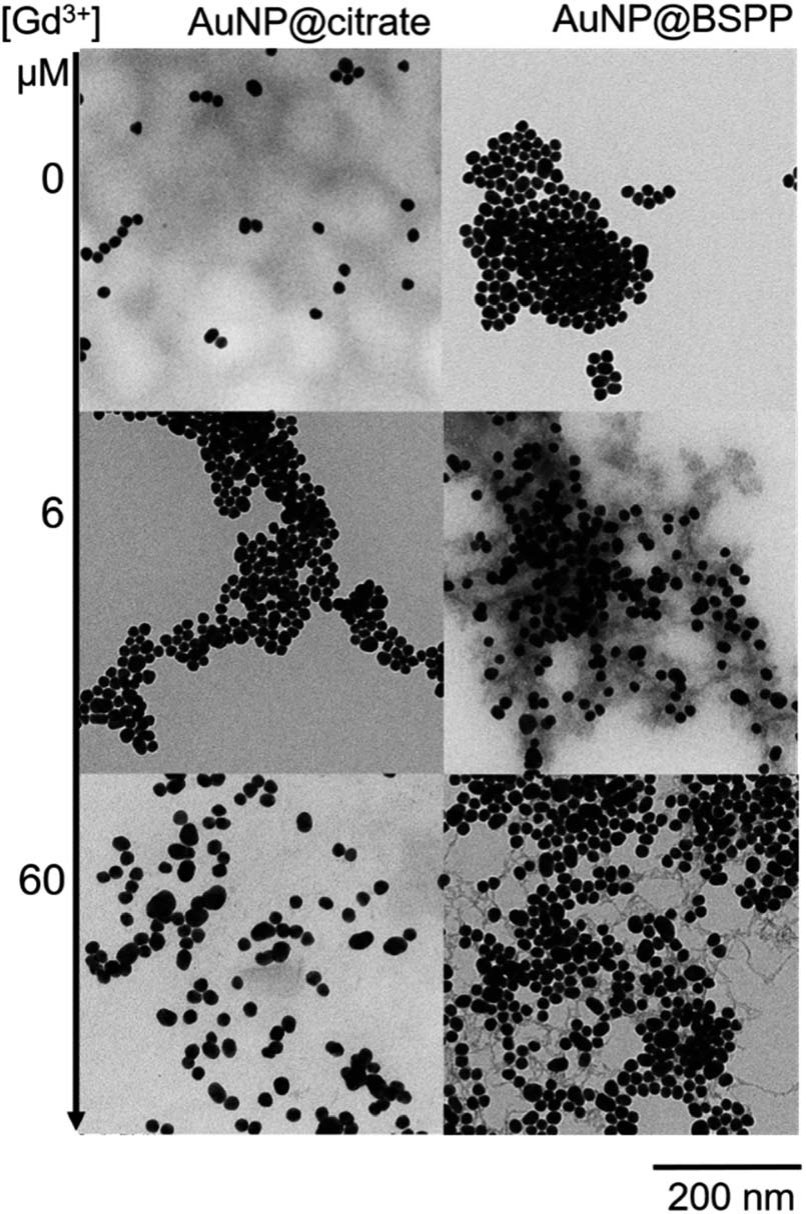
TEM images of AuNP@citrate (left) and AuNP@BSPP (right) before and after incubation with 6 and 60 μM of Gd(NO_3_)_3_, respectively.

Transmission Electron Microscopy (TEM) images realized of AuNP@BSPP after the addition of two different concentrations of Gd(NO_3_)_3_: 6 μM (for the slow aggregation regime) and 60 μM (for the fast one) are shown in Fig. 9 and ESI Fig. S12.† The aggregation of the NPs observed on the TEM images cannot be unambiguously interpreted due to drying-artifacts coming from the TEM sample preparation. However, a grey shell connecting the NPs is observed on both samples. This shell connects the NPs and is also found in their immediate surroundings. As investigated by Schubert *et al.*,^43^ this shell can be attributed to the presence of Gd hydroxides and can be considered to be an argument for the aggregation of the nanoparticles *via* interactions of sulfonate groups with the Gd^3+^ ions without replacement of the ligands from the first coordination sphere of Gd. Together, reversibility of the interactions observed in QCM-D experiments and the possibility to reverse the particle aggregation by the addition of a strong coordinating ligand for the Gd^3+^ ions, *i.e.* ethylenediamine tetra(methylene phosphonic acid) (EDTMP) (see ESI Fig. S13†) account for the labile character of the Gd^3+^ sulfonate interactions.

**Fig. 10:**
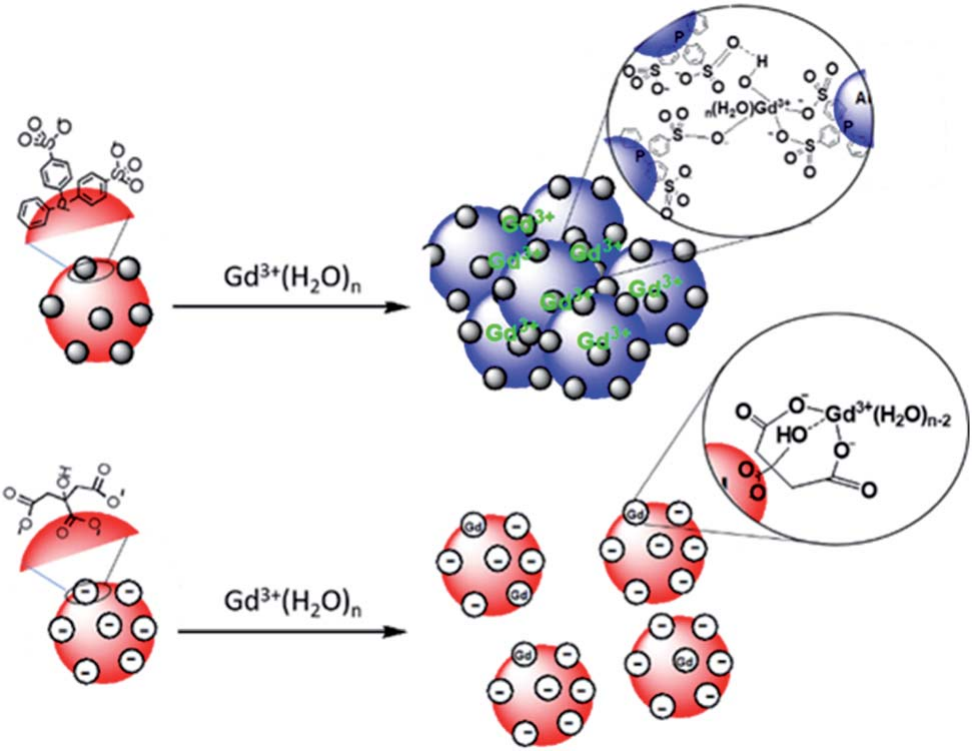
Schematic representation of the interaction of Gd^3+^ ions with the surfaces of AuNP@BSPP and AuNP@citrate, respectively.

Regarding the AuNP@citrate system, no change in the value of *ζ*-potential and in pH (*i.e.* 5.7), and thus, on the colloidal stability was evidenced after the addition of 25 μM of Gd^3+^ ions. Besides, no grey shell was detected on the TEM image (Fig. 9). The free carboxylic groups of the citrate ligand can now directly bind to the Gd^3+^ ions in the first coordination sphere of the ion by replacing water and/or hydroxy groups.^40^ From the literature results, this complex can be mainly described as a 1 : 1 complex between Gd^3+^ and citrate molecules.^40^ Hence, as the concentration of Gd^3+^ ions used in the experiments depicted in Fig. 3 is low compared to the concentration of citrate present in solution (about 10 times lower), the formation of 1 : 1 complexes with either the citrate in interaction with the surface of AuNPs or with the citrate free in solution is favored.^48^ Au surfaces retain their negative net charge as shown by *ζ*-potential measurements. This further prevents the aggregation process of AuNP@citrate solution in the presence of Gd^3+^ ions and makes the system unsuitable for the implementation of a colorimetric sensing test. On the contrary, the interaction between the sulfonate groups and the Gd^3+^ ions makes the AuNP@BSPP system extremely sensitive and suitable for the development of a cost-effective, fast and easy bench colorimetric assay (see Fig. 10).

### AuNP@BSPP as a colorimetric sensing probe

**Fig. 11:**
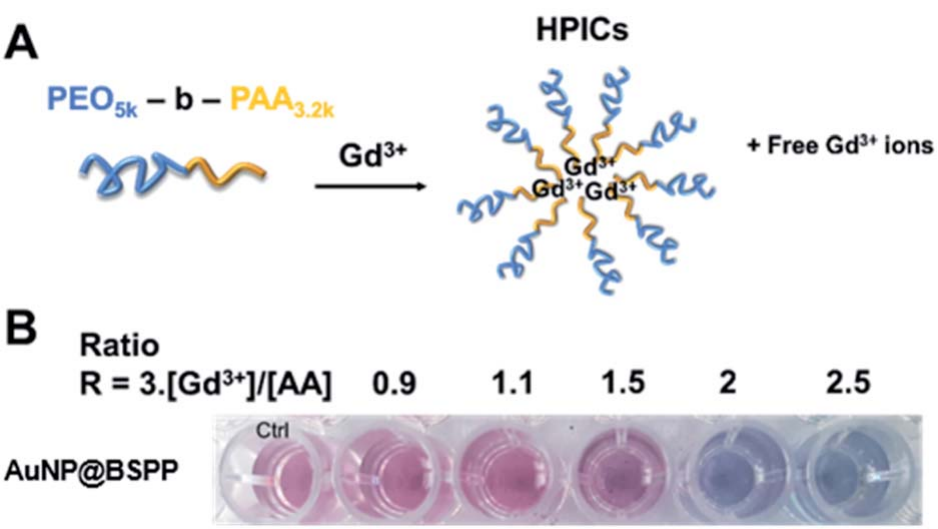
(A) Representation of the mechanism of formation of Hybrid Polyion Complexes (HPICs) with excess of gadolinium ions. (B) Photographs showing the color response of AuNP@BSPP to increasing ratios *R* of positive charges over negative charges.

Based on the presented results, AuNP@BSPP would allow the quantification of free Gd^3+^ ions in solutions at a neutral or slightly lower pH in order to avoid the formation of low soluble gadolinium hydroxides and oxides. The assessment of this method was performed through the preparation of a Gd-based contrast agent used in magnetic resonance imaging (MRI). As stated above, the precise evaluation of such free ions is critical as for *in vivo* applications their level needs to be equal to zero to avoid toxicity issues.^49^ MRI contrast agents, based on the complexation of Gd^3+^ ions, with a double-hydrophilic block copolymer made of poly(acrylic acid), PAA, and poly(ethylene oxide) PEO blocks were formed. As illustrated in Fig. 11A the ratio of positive charges (coming from gadolinium) over negative charges (coming from PAA) influences the formation of hybrid polyion complexes (noted HPICs) and leads to a possible *exo* gadolinium quantity corresponding to the “excess” of positive charges introduced in the system. Five different HPICs with five different charge ratios (Gd^3+^/PAA): 0.9, 1.1, 1.5, 2 and 2.5 were prepared here according to the protocol detailed in the ESI.† The obtained colloids have a mean hydrodynamic diameter around 20 nm as determined by dynamic light scattering. These colloids exhibit exceptionally high stability upon dilution and generate high water proton relaxivities *in vitro* and an excellent tolerance *in vivo* after intra-venous injection on a rat model with a resulting positive signal enhancement.^50^

A colorimetric test using AuNP@BSPP was first applied as shown in Fig. 11B. As expected for the lower ratio 0.9, no color change was observed, whereas a ratio higher than 1.1 led to a change of observed color suggesting the presence of free Gd^3+^ ions.

Beyond this qualitative evaluation, quantitative measurements based on UV-visible spectroscopy (ESI Fig. S15†) and on the calibration curve presented in Fig. 3B were further performed. For this, the five solutions of HPICs were filtrated to separate free Gd^3+^ ions from obtained HPICs. Filtrates were then taken up to be used as samples for the colorimetric test. The first two filtrates, at ratios 0.9 and 1.1 do not contain any quantifiable amount of Gd^3+^. For the charge ratio 1.5, an estimation of Gd^3+^ free ion content of 13.2 ± 0.8 μM is measured, in good agreement with the 12 μM expected value.

Evaluation of the Gd^3+^ ions content for the two highest charge ratios is not relevant as the obtained values are not in the linear range determined previously. The determination of free Gd^3+^ ions through the use of the FP was found to be more precise than the one obtained by calculating the ratio of absorbance measured at two different wavelengths at 524 and 640 nm, respectively as proposed in the literature (see ESI Fig. S16† for comparison).^3,51^ The estimated value compares very favorably with the one estimated from ICP measurements 9.8 ± 0.5 μM with a higher accuracy. Hence, the application of the colorimetric test and the aggregation index method has proved to provide a good estimation of the concentrations of free gadolinium ions. The limits and ranges of detection of AuNP@BSPP are comparable with the ones obtained by conventional methods like chromatographic techniques coupled either with spectroscopic or with ICP techniques (see Table 1). Despite their simplicity, high sensitivity and convenience, few are the colorimetric tests based on AuNPs designed for the detection of lanthanide ions,^3^ thus justifying the importance to develop a colorimetric test based on AuNP@BSPP.

**Table 1:** Values of limits and range of detection of gadolinium obtained from UV-visible AuNP@BSPP detection (present work) and from conventional methods found in the literature

Analysis techniques	Gadolinium specie	Linear or fitting range of concentrations	Limits of detection	Limits of quantification	Ref.
UV-vis	Gd^3+^	0–18 μM	0.74 μM	4.76 μM	This paper
MEKC^a^/UV-vis	Gd-based contrasting agents	5–200 μM or 100–5000 μM according to Gd CAs	0.40–20 μM	N.P.^b^	52
HPLC^c^/UV-vis	GdDTPA-BMA	2–800 μM (serum), 10–2000 μM (urine)	0.3 μM (serum), 1.1 μM (urine)	2 μM (serum), 10 μM (urine)	53
HPLC^c^/ICP-AES^d^	Gadodiamide	0–590 μM (ICP-AES of serum)	1.9 μM (ICP-AES of serum)	6.5 μM (ICP-AES of serum)	54
ESI-MS^e^/ICP-OE^f^	Gd-based contrasting agents	5–100 μM	0.1–1 μM	0.5–5 μM	55
HPLC^c^/ICP-OES^f^	Gd-based contrasting agents	2.5–500 μM	0.05–0.2 μM	0.16–0.73 μM	56

a MEKC (Micellar Electrokinetic Capillary Chromatography).

b N.P. = Not Precised.

c HPLC (High Performance Liquid Chromatography).

d ICP-AES (Inductively Coupled Plasma Atomic Emission Spectroscopy).

e ESI-MS (Electrospray Ionization-Mass Spectrometry).

f ICP-OES (Inductively Coupled Plasma Optical Emission Spectroscopy).

## Experimental

### Materials

HAuCl_4_·3H_2_O (≥99.9%) was purchased from Alfa Aesar. Tri-sodium citrate dihydrate (>99%), bis-(*p*-sulfonatophenyl)phenylphosphine dehydrate dipotassium salt (97%) and all the metal salts were purchased from Sigma-Aldrich. The solutions of electrolytes were prepared from NaCl, Ca(NO_3_)_2_·4H_2_O, and Gd(NO_3_)_3_·6H_2_O by separately dissolving each compound in water. Water was purified through a filter and ion-exchange resin using a Purite device (resistivity 18.2 MΩ cm).

### Synthesis of AuNP@citrate nanoparticles

All glassware and magnetic stir bars used for gold nanoparticle synthesis were carefully washed with aqua regia (HCl/HNO_3_ 3 : 1, v/v) and abundantly rinsed in distilled water. Since HAuCl_4_ is corrosive, a glass spatula was used in order to avoid contact with the metal. Gold nanoparticles with a diameter of 17 nm were prepared following the procedure of Turkevich.^6^ 10 mL of a solution of sodium citrate tribasic trihydrate (17 mmol L^−1^) was added to 180 mL of a boiling aqueous solution of HAuCl_4_ (0.3 mmol L^−1^). The reaction mixture was maintained at 100 °C for 30 minutes under magnetic stirring before allowing it cool down to room temperature. A color change from pale yellow to blue and dark red in the end of the reaction was observed.

### Synthesis of AuNP@BSPP nanoparticles

AuNP@citrate were further functionalized with dipotassium bis(*p*-sulfonatophenyl)phenylphosphine dihydrate following a literature procedure.^12^ For a typical reaction, 100 mL of AuNP@citrate solution was stirred with the sulfonated phenylphosphine ligand (30 mg, 0.06 mmol) for one night. The AuNP@BSPP were isolated by adding solid NaCl until the color changed from dark red to blue, followed by centrifugation (2000 rpm for 5 minutes at 20 °C). The isolated particles were then redispersed in a solution of sulfonated phenylphosphine (0.5 mmol L^−1^). A new cycle of precipitation was performed by adding methanol (about 6 mL), followed by their redispersion in the sulfonated phenylphosphine solution. In this way the AuNP@BSPP particles were cleaned from the citrate ligand and can be concentrated to give any desired concentration.

### UV-visible spectroscopy

UV-visible spectra were measured with a SPECTROstar Nano® – BMG Labtech absorbance plate reader. Multi-well plates with 96 well-cell culture plates, sterile F-bottom type, from Cellstar ® – Greiner bio-one were used. For the measurements the wells were loaded with a total volume of 200 μL.

### Multi-well plate preparation

Each well of the plate supposed to be measured was filled with a maximum volume of 200 μL. For a given line, the first well was associated with the blank (water) and the second with a standard corresponding either to the AuNP@citrate or to the AuNP@BSPP at the same concentration (0.14 mM in gold atoms). For the next wells, mixtures of 2 or 3 components were made, in the following way of addition (1) water, (2) metallic salt solution (except for standard well), (3) AuNP solution. Concentrations of the AuNPs were checked through UV-visible spectroscopy of the solution before micro-plate elaboration, by evaluating the optical density for the maximum absorbance value. Metallic salt solutions were added in order to obtain increasing concentration from one well to another. UV-visible spectroscopy analysis was conducted at 15 min after addition of the AuNPs in the wells and recorded every 15 minutes for 4 hours.

### Dynamic light scattering (DLS) and zeta potential measurements

Measurements of the size and the *ζ* potential of the particles were realized with a Malvern Zetasizer NanoZS instrument. Parameters of the measurements were set on Au type of particles. Measurements were realized at 25 °C and 5 series of 10 recorded signals of 10 seconds were realized for size data records and only 3 series for zeta potential data. The DLS data were analyzed using the general-purpose non-negative least-squares (NNLS) method.

### Quartz crystal microbalance with dissipation monitoring (QCM-D)

QCM-D measurements were performed on a QCM-D QSense Explorer device with one sensor at the AIME platform from INSA Toulouse. AT-cut gold-coated quartz crystals with a fundamental resonance frequency of 10 MHz were purchased from QSense Biolin Scientific. All experiments were conducted at 20 °C and a flow rate of 40 μL min^−1^. The frequency shifts (Δ*F*) and the dissipation coefficient (Δ*D*) were recorded as a function of time at different overtones (1, 3, 5, 7, 9, 11, and 13). A sensor chamber was incubated with citrate or BSPP solutions overnight (∼16 hours). The data were collected and analyzed with QSoft and QTool software.

In a QCM-D experiment, the adsorption of molecules on the gold surface is assessed by monitoring the frequency shift (Δ*F*) and dissipation shift (Δ*D*) (the frictional and viscoelastic energy losses in the system) as a function of time. The changes in frequency are proportional to the changes in the wet-mass deposited on the gold surface according to the Sauerbrey equation^57^ (eq (2)):2
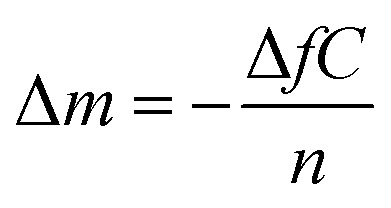
where Δ*m* is the adsorbed mass, Δ*f* the variation of frequency observed at overtone *n* and *C* = 17.7 ng cm^−2^ a characteristic constant of the instrument. Even if this model only applies to uniform and rigid thin film deposits, it allows a simple quantitative study and a direct comparison of experiences.

### Transition electronic microscopy (TEM)

TEM images were acquired on a Hitachi HT7700 device in collaboration with the platform CMEAB from Toulouse. Samples were prepared by removing the charge from the carbon coated copper grids (CF200-Cu 200 mesh from Tel Pella Inc.) and then depositing them on a drop of the solution to analyze, on the shiny side. After 5 minutes, the residual solution was removed by blotting with a filter paper.

## Conclusions

In this paper, we evidenced for the first time the interaction between Gd^3+^ ions and the negatively charged surface of AuNP@BSPP bearing sulfonate groups. AuNP@BSPP were obtained after ligand exchange of BSPP with AuNP@citrate obtained by Turkevich method. AuNP@BSPP present high stability and can interact with different families of ions; among them gadolinium is of special interest. These interactions resulted in a colour change that induced the aggregation of AuNP@BSPP even in the presence of very low concentrations of Gd^3+^ ions (*i.e.* μM range). As demonstrated by UV-visible spectroscopy, QCM-D measurements and electronic microscopy, the mechanism of interactions occurred thanks to reversible interactions between the Gd^3+^ ions and the sulfonate groups surrounded by a water rich coordination sphere. These results were compared with the ones obtained with AuNP@citrate for which no aggregation was observed in the same range of Gd^3+^ ion concentrations due to strong specific interactions of Gd^3+^ ions with citrate moieties *via* a 1 : 1 complex. We took advantage of this phenomenon to develop a simple and fast bench colorimetric assay for the detection of free Gd^3+^ ions based on the determination by UV-visible measurements of the flocculation parameter. This one when represented as a function of gadolinium concentrations can be used as a calibration line with a linear range from 0 to 18 μM. Limits of detection and quantification were found equal to 0.74 μM and 4.76 μM of Gd^3+^ respectively. Finally, this colorimetric assay was successfully used to probe the quantity of remaining free Gd^3+^ ions present in solution following the preparation of Gd-based contrast agents for magnetic resonance imaging through complexation of gadolinium with polymers as hybrid polyion complexes. Our work is of particular importance as the proposed method is cost-effective, simple to implement with limits and ranges of detection comparable with the most conventional methods which in return are more time consuming, costly and difficult to implement.^2^ Additionally, in comparison with other colorimetric tests based on AuNPs,^58^ the AuNP@BSPP system does not require any special design of the ligand before functionalization. The BSPP ligand is commercially available and the functionalization of the gold nanoparticles is reproducible and easy to perform. AuNP@BSPP are robust and have already been used as a platform for further functionalization.^9,10^ It could thus lead to the generalization of this test in the laboratories working in the development of gadolinium complexes for a rapid on-site detection of free Gd^3+^ ions.

## Conflicts of interest

There are no conflicts to declare.

## Supplementary Material

NA-002-D0NA00374C-s001
