# Supplementary material for: Easy colorimetric detection of gadolinium ions based on gold nanoparticles: key role of phosphine-sulfonate ligands
Source: Nanoscale Adv. 2020 Sep 7;2(10):4671–81. doi: 10.1039/d0na00374c (PMC9417556; doi:10.1039/d0na00374c)
Supplement: NA-002-D0NA00374C-s001 [file NA-002-D0NA00374C-s001.pdf]

Electronic Supplementary Material (ESI) for Nanoscale Advances.
This journal is © The Royal Society of Chemistry 2020

Supporting Information

Easy colorimetric detection of Gadolinium ions based on gold nanoparticles: Key role of phosphine-sulfonate ligands.

Marjorie Yon,[a] Claire Pibourret [a] Jean-Daniel Marty*[a] and Diana Ciuculescu-Pradines*[a]

Table of Contents

1. **Determination of the size of AuNP@citrate from UV-visible spectra**
2. **TEM of AuNP@citrate**
3. **Determination of the hydrodynamic size of AuNP@citrate and AuNP@BSPP**
4. **Sedimentation Field-Flow Fractionation**
5. **Zeta potential**
6. **Colour response of AuNP@citrate and AuNP@BSPP after incubation with increasing concentrations of $Gd(NO_3)_3$**
7. **UV-visible spectra of Gd^{3+}-AuNP@BSPP, registered each 15 minutes for 4h of time of incubation**
8. **UV-visible spectra of Gd^{3+}- AuNP@citrate, registered at t=0h (A) and at t=4h (B)**
9. **Flocculation parameter**
10. **Photographs showing the colour response of AuNP@citrate and AuNP@BSPP after incubation with increasing concentrations of different metallic salts.**
11. **QCM-D measurements**
12. **TEM images of AuNP@BSPP and AuNP@citrate after incubation with Gd^{3+} ions**
13. **Evidence of the reversibility of the aggregation of AuNP@BSPP by Gd^{3+} ions**
14. **Synthesis of Hybrid Polyion Complexes (HPICs)**
15. **UV-visible spectra of AuNP@BSPP in the presence of the filtrates resulted from the purification of HPICs**
16. **Determination of the free Gd^{3+} ions concentration through the ratio of absorbance A_{640}/A_{524}**

1. Determination of the size of AuNP@citrate from UV-Vis spectra.

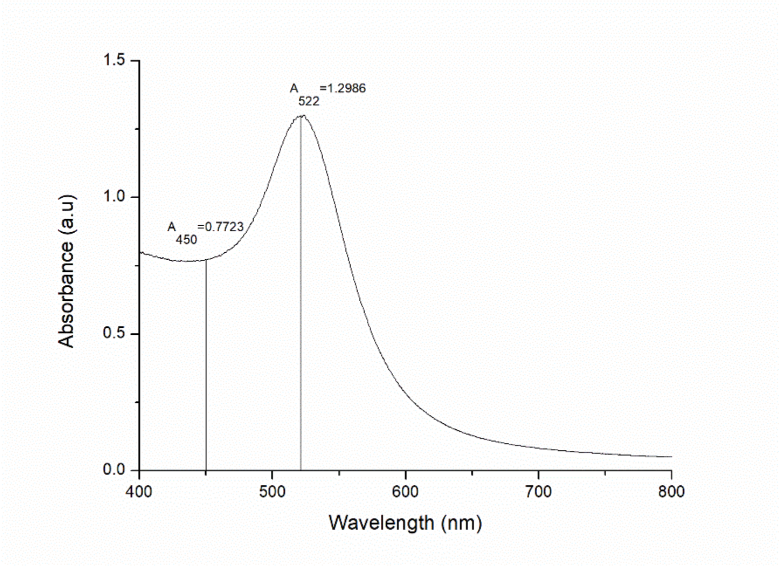

Fig. S1 UV-visible spectra of AuNP@citrate

A_{spr}/A_{450}=1.2986/0.7723=1.68

Table. S1 Ratio A_{spr}/A_{450} in dependence of the particle diameter from ref. 1

A_{spr}/A_{450}	d(nm)
1.65	16
1.69	18

2. TEM of AuNP@citrate

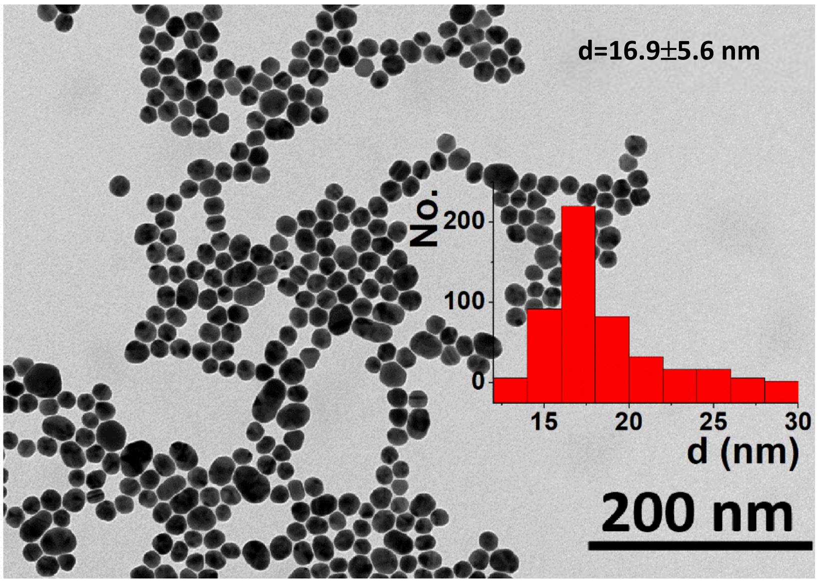

Fig. S2 TEM micrograph of AuNP@citrate and corresponding size distribution (inset).

3. Determination of the hydrodynamic size of AuNP@citrate and AuNP@BSPP.

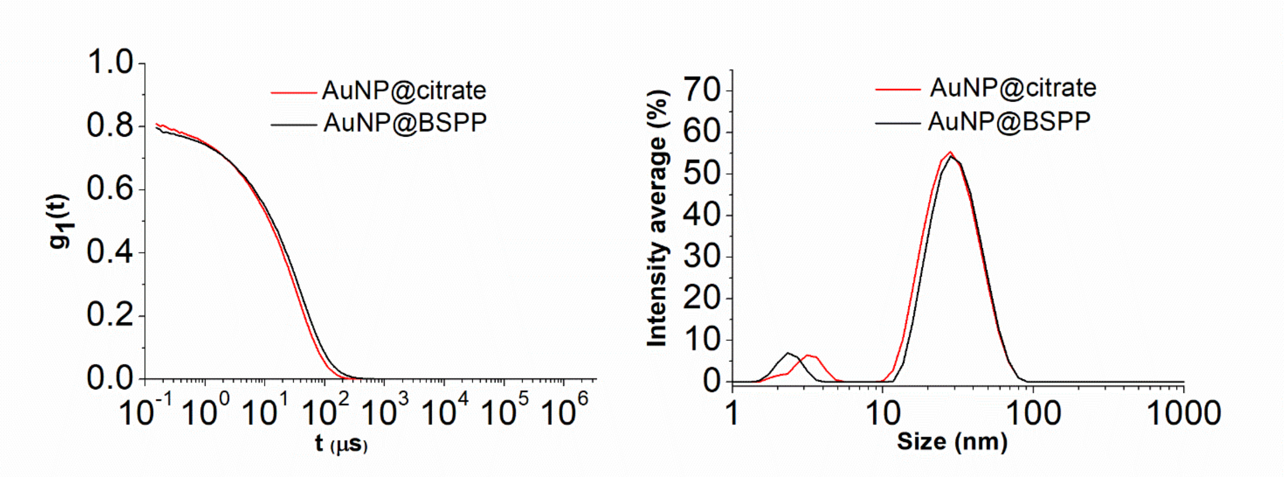

Fig. S3 Correlogram functions (left) and intensity-averaged hydrodynamic diameter distribution (right) estimated from dynamic light scattering measurements of AuNP@citrate (red curve) and AuNP@BSPP (black curve) for a gold ions concentration of ca. 0.28 mM.

4. Sedimentation Field-Flow Fractionation:[2]

Sedimentation Field-Flow Fractionation (SdFFF) belongs to the Field-Flow Fractionation (FFF) family, an ensemble of separation techniques similar to chromatographic methods, suitable for separating and determining the mass (or size) of colloidal particles with high accuracy. The method is very reproducible. The separation process occurs in a ribbon like channel in the absence of any stationary phase, so that the degree of irreversible adsorption is considerably reduced.

SdFFF analyzes were carried out at the TFFFC platform (Toulouse Field Flow Fractionation Center, INP Purpan) on the CF2000 Centri FFF device from Postnova with a UV-visible detector (λ = 254, 520 or 620 nm). The samples were injected via a 21.8 µL Rheodyne valve. The channel was 250 µm thick. The eluent used was ultrapure water (18MΩ.cm from MiliQ Water purification system), with approximately 18 µM of BSPP for the elution of AuNP@BSPP and 30 µM sodium citrate for the elution of AuNP@citrate, respectively. The parameters used were as follows: a flow of 1 mL*min^{-1}, a detection sensitivity at 2.10^{-4} absorption units and a centrifugation cycle defined as in Fig. S4A.

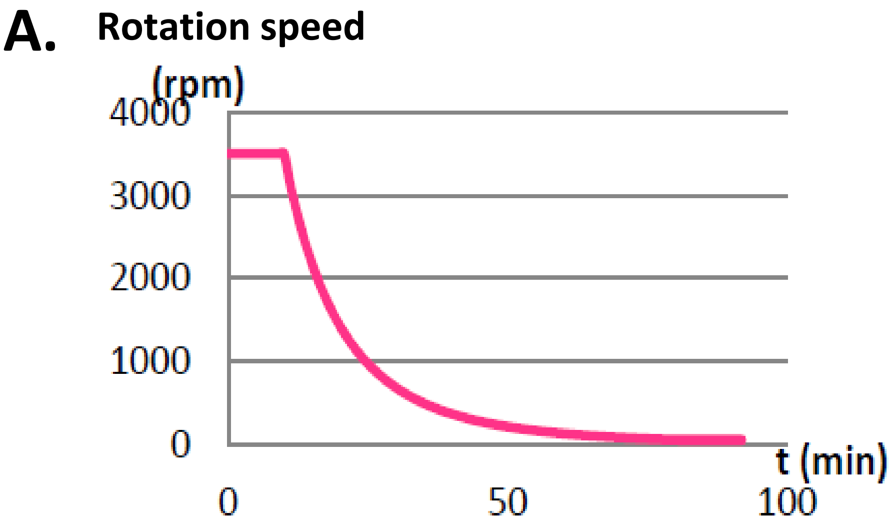

Fig. S4A The rotations set to perform the fractionations

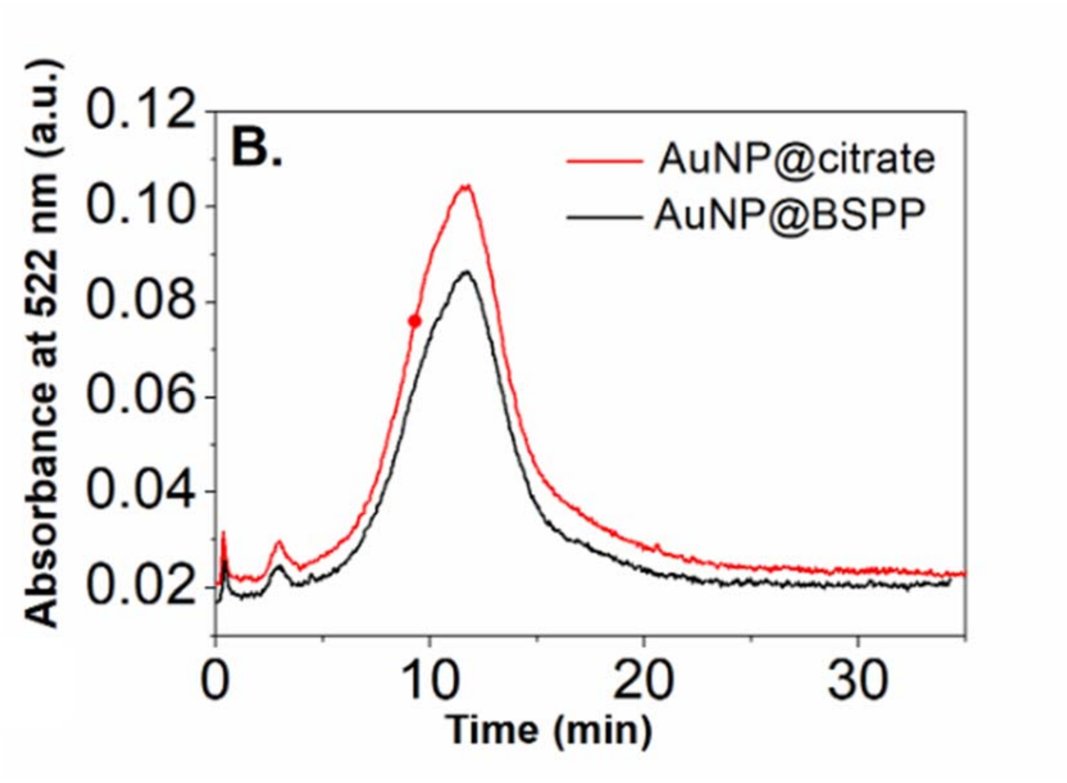

Fig. S4B SdFFF chromatogram of AuNP@citrate and AuNP@BSPP, respectively.

5. Zeta potential

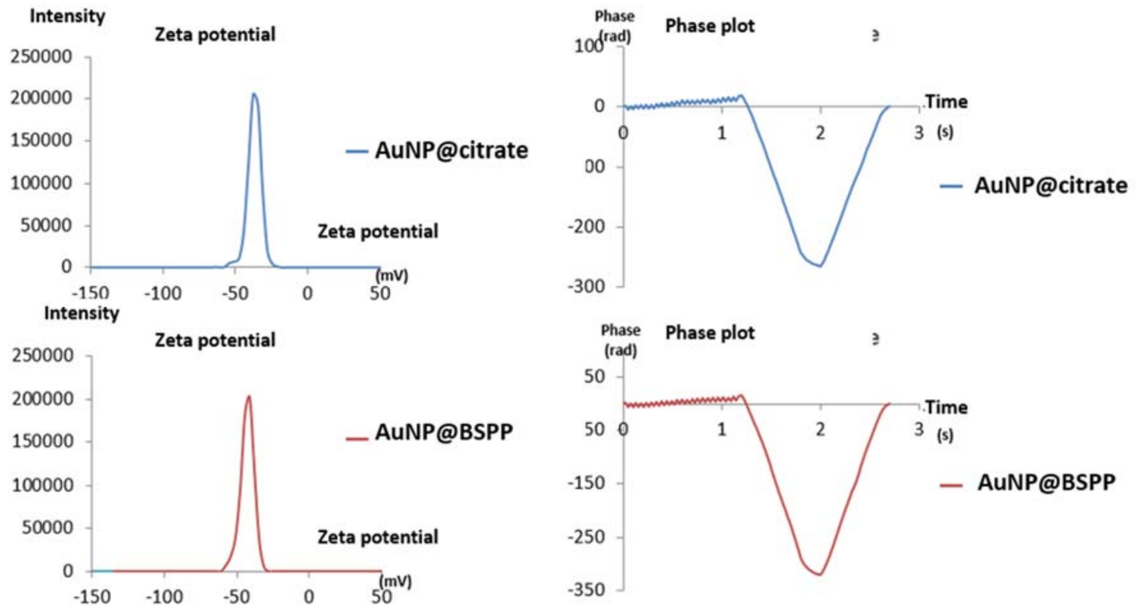

Fig. S5 Zeta potential and corresponding phase plot for AuNP@citrate and AuNP@BSPP for a gold ions concentration of ca. 0.28 mM.

6. Colour response of AuNP@citrate and AuNP@BSPP after incubation with increasing concentrations of $Gd(NO_3)_3$.

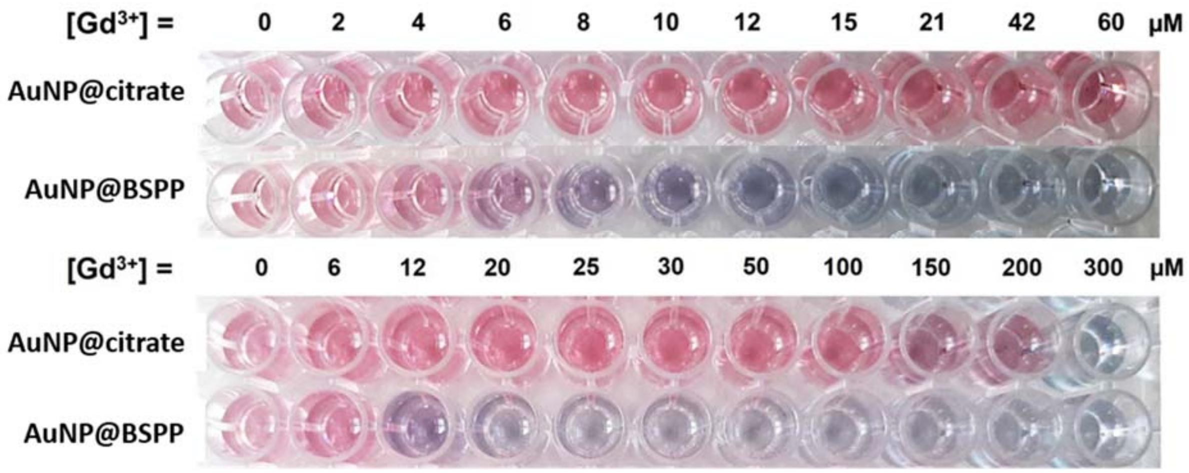

Fig. S6 Photographs showing the colour response of AuNP@citrate and AuNP@BSPP after incubation with increasing concentrations of $Gd(NO_3)_3$.

7. UV-visible spectra of Gd^{3+}-AuNP@BSPP, registered each 15 minutes for a period of 4h of time of incubation.

The first spectra was registered at 15 minutes after the addition of the Gd^{3+} ions. On the graphs at t=0, the spectra of the AuNP@BSPP without any addition of Gd^{3+} ions was represented.

A .

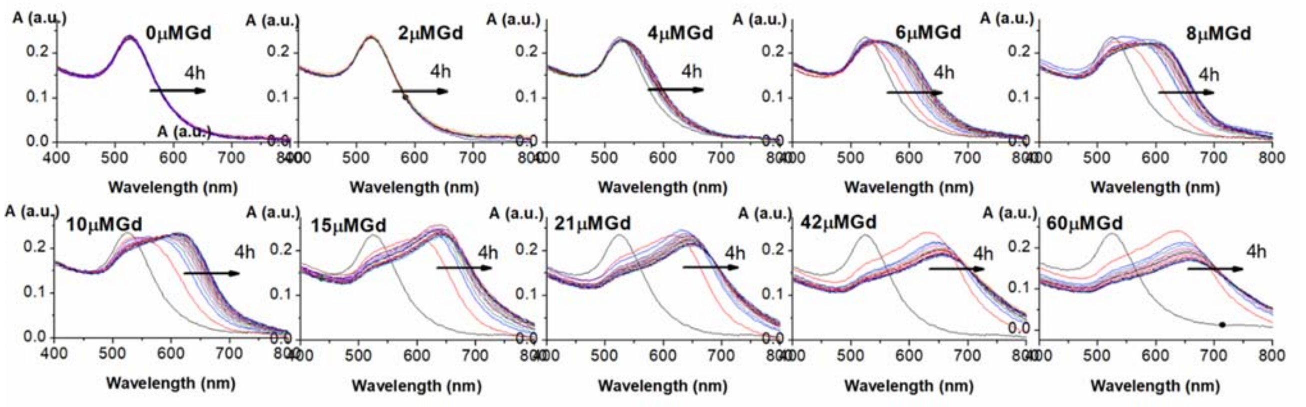

B.

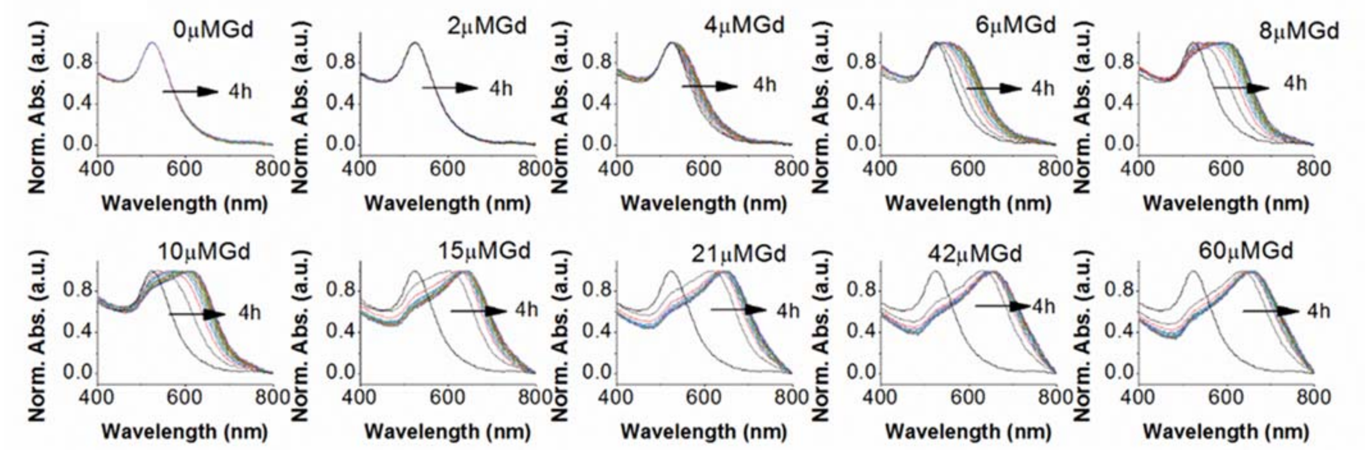

Fig. S7 Evolution of the UV-visible spectra as a function of time for AuNP@BSPP upon addition of increasing concentrations of $Gd(NO_3)_3$ (measurement time interval 15 minutes for a period of 4h). AuNPs concentration is ca. 0.14 mM estimated in gold ions. (A) Brut absorbance spectra and (B) Normalized absorbance spectra.

8. UV-visible spectra of Gd^{3+}- AuNP@citrate, registered at t=0h (A) and at t=4h (B)

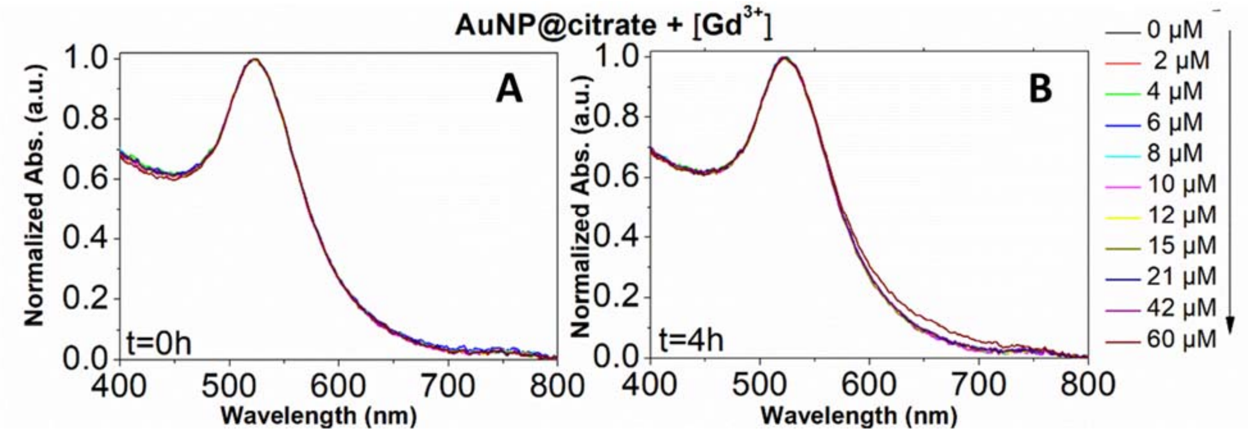

Fig. S8: Evolution of UV-visible spectra of AuNP@citrate in presence of increasing concentrations of $Gd(NO_3)_3$ at t=0h and t=4h for a gold ions concentration of ca. 0.14 mM.

9. Flocculation parameter and statistics

The flocculation parameter was defined as follows. First, the UV-visible spectra of the Au nanoparticles and those registered at different ionic strengths of $Gd(NO_3)_3$ and different times of incubation are normalized to the intensity of the surface plasmon resonance wavelength. Then, the integrated absorption between wavelength limits 600 and 800 nm of original spectra of gold nanoparticles was subtracted from the one calculated for the spectra of interest, between the same limits.

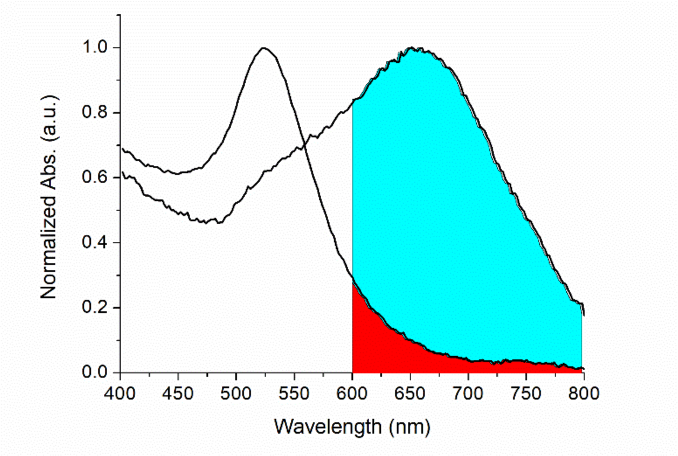

Fig. S9 Normalized UV-visible spectra of AuNP@BSPP after and before the incubation with 60 µM of $Gd(NO_3)_3$ and the representation of the corresponding integrated areas for the subsequent calculation of the flocculation parameter. The blue part corresponds to the flocculation parameter.

As represented in Figure 4B and below, FP vs. [Gd^{3+}] presents a linear range from 0 to 18 µM. The equation of the calibration curve is given by:

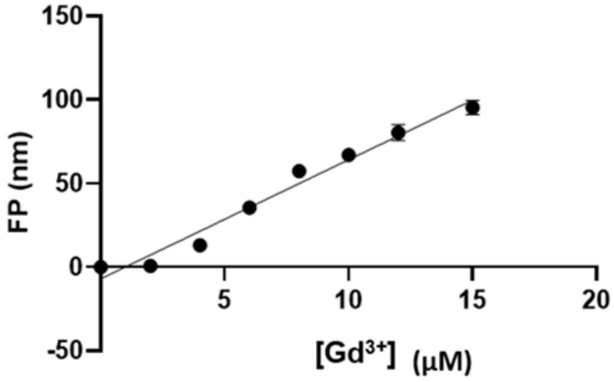

$FP = a + b \times [Gd^{3+}]$

with a linear correlation coefficient r^2 equal to = 0.974

$a = -6.994$ and $s_a = 4.096$

$b = 7.118$ and $s_b = 0.478$

Limits of detection and of quantification were calculated from these values as

$[Gd^{3+}]_{LD} = (a + 3 \times s_a)/b = 0.74$ µM and $[Gd^{3+}]_{LQ} = (a + 10 \times s_a)/b = 4.76$ µM

$FP_{LD} = a + 3 \times s_a = 5.27$ Abs.nm and $FP_{LQ} = a + 10 \times s_a = 33.90$ Abs.nm

The coefficient of variation (CV) of repeatability was calculated from the repetition of nine measurements that lead to the determination of the respective FP values: 22.77, 21.22, 21.89, 22.80, 20.78, 22.11, 24.83, 18.14 and 19.99. Mean = 21.62 Abs.nm and corresponding standard deviation equal to 1.90. CV= 1.90/21.62*100=8.8%.

The standard deviation on gadolinium concentration values obtained from calibration curve for a sample with unknown concentration of gadolinium was evaluated by using the following equation:

$$s_c = \frac{s_r}{b}\sqrt{\frac{1}{M} + \frac{1}{n} + \frac{(\overline{y_c} - \overline{y})^2}{b^2 . \Sigma (x - \overline{x})^2}}$$

with M the number of repetitions for the unknown sample, n the number of points used to establish the calibration curve (here n=9), y and x represent the coordinates of the point in the calibration line, $s_r^2 = s_b^2 * (\sum(x-\langle x \rangle)^2$, y_C is equal to the value of FP estimated on the sample with unknown concentration of gadolinium.

10. Colour response of AuNP@citrate and AuNP@BSPP after incubation with increasing concentrations of different metallic salts.

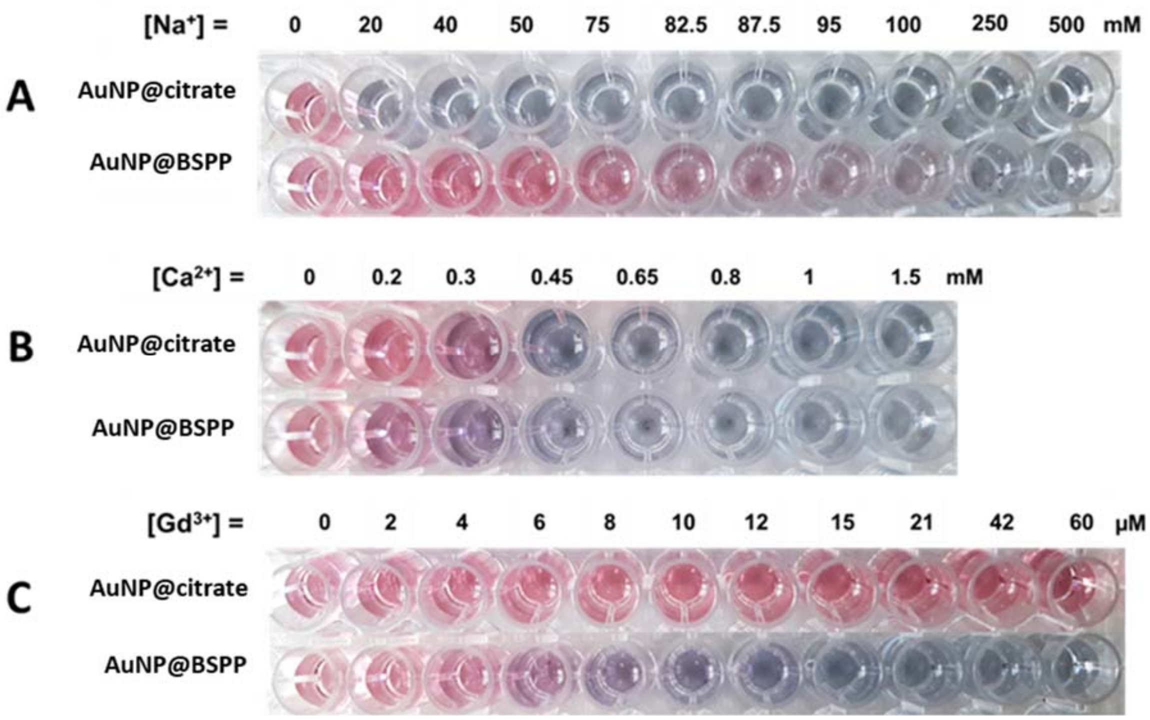

Fig. S10 Photographs showing the colour response of AuNP@citrate and AuNP@BSPP after incubation with increasing concentrations of NaCl (A), $Ca(NO_3)_2$ (B) and $Gd(NO_3)_3$ (C), respectively.

11. QCM-D measurements

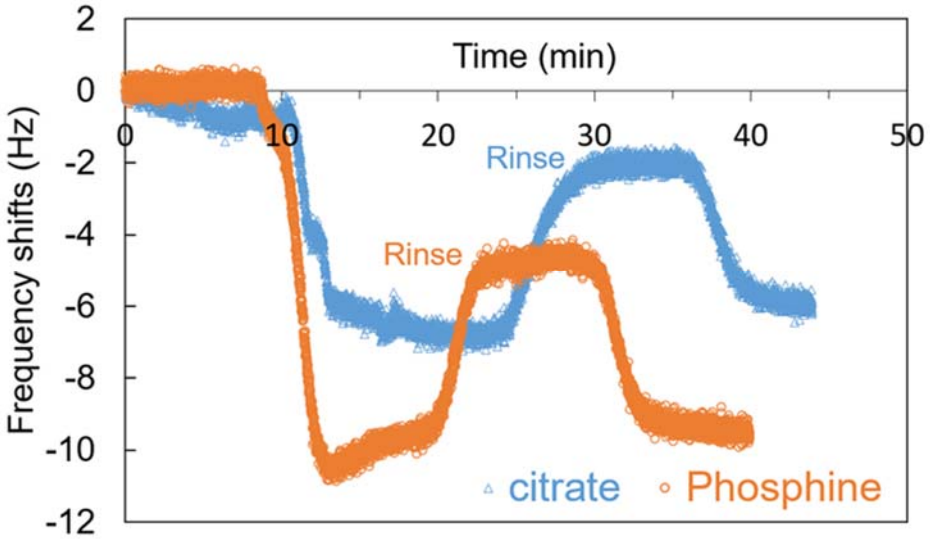

Fig. S11.1 QCM-D measurements showing the shift in frequency with time for the deposition onto a gold surface of the citrate (24mM) and the phosphine BSPP (24mM), respectively. After the steady-state was reached, the substrate was rinsed with water in order to wash out the reversible attached molecules.

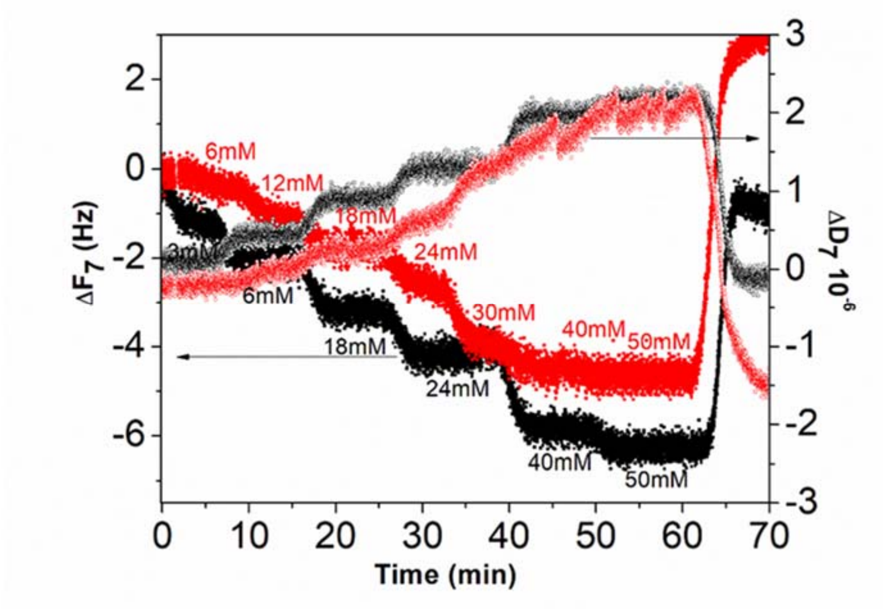

Fig. S11.2 QCM-D measurements showing the shifts in frequency and dissipation with time for the adsorption of Gd^{3+} ions onto precoated gold surfaces. The measurements were carried out as sequential deposition of increasing concentrations of Gd^{3+} ions. Red curve refers to the experiments carried out on a citrate precoated gold QCM-D surface and the black one of the ones carried out on the BSPP precoated surface, respectively.

11.3 Langmuir and Langmuir-Freundlich model

The Langmuir model assumes that the adsorption energy of active sites is always similar, and adsorption occurs in a monolayer way.[3] The Langmuir model is described by eq 1:

$$\Gamma = \frac{\Gamma_{max} K_L [Gd^{3+}]}{1+K_L[Gd^{3+}]} \qquad \text{(eq 1)}$$

Where:
Γ**max** is the maximum surface coverage of gadolinium ions (nmol/cm^2)
[Gd^{3+}] is the gadolinium ions concentration (mM)
K_L is the binding Langmuir constant that corresponds to the inverse of the Gd^{3+} concentration that is required to obtain one-half of Γmax (mM^{-1})

Langmuir-Freundlich equation[4]

$$\Gamma = \frac{\Gamma_{max*} (K_{LF}[Gd^{3+}])^n}{1+(K_{LF}[Gd^{3+}])^n} \quad \text{(eq 2)}$$

Where:
Γ**max** is the maximum surface coverage of gadolinium ions (nmol/cm^2)
[Gd^{3+}] is the gadolinium ions concentration (mM)
K_{LF} is the binding Langmuir-Freundlich constant that corresponds to the inverse of the Gd^{3+} concentration that is required to obtain one-half of Γmax (mM^{-1})
n Langmuir-Freundlich coefficient, and represents the degree of non-linearity

When n = 1, eq. 2 converts to the Langmuir model. For n > 1, eq. (2) may be identified as describing a cooperative reaction between the sorption site and n sorbate molecules.

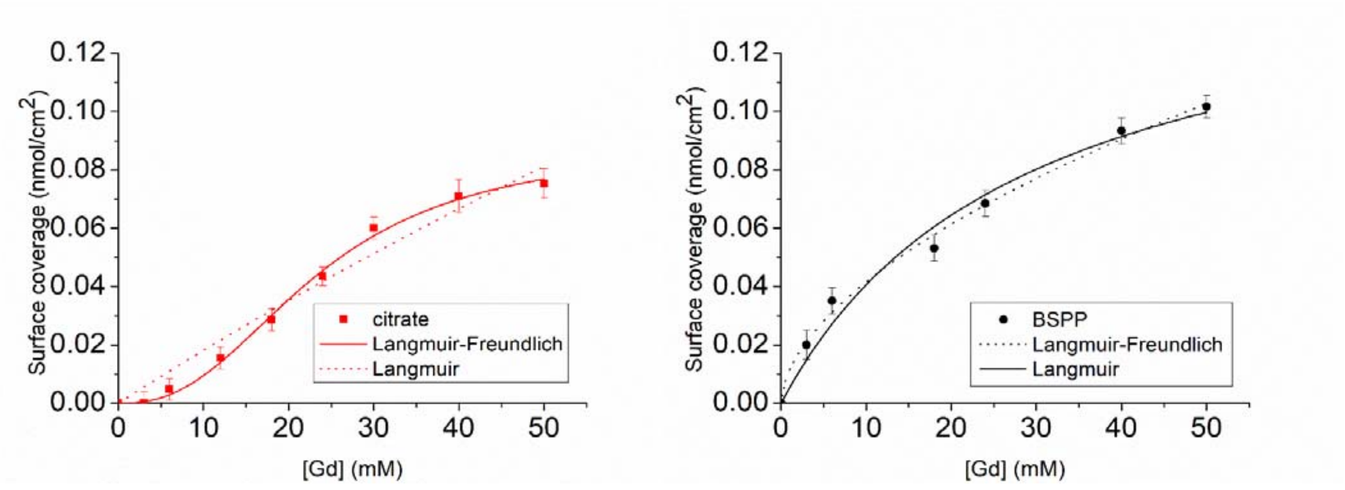

Fig. S11.3 Plots of the surface coverage *versus* concentration of Gd^{3+} ions, fitted using both Langmuir and Langmuir-Freundlich model. On the left for the citrate precoated surface and on the right for the BSPP precoated substrate.

Isotherm model	Parameters Gd^{3+} adsorbate	Coated QCM-substrate	
		citrate	phosphine
Langmuir	$\Gamma_{max(L)}$ (nmol/cm^2)	-	0.15±0.02
	K_L (mM^{-1})	-	0.034±0.010
	R^2	-	0.97
Langmuir-Freundlich	$\Gamma_{max(LF)}$ (nmol/cm^2)	0.088±0.005	40.70±7387.65
	K_{LF}(mM^{-1})	0.042±0.002	5.10*10^{-7}±1.64*10^{-4}
	n	2.5±0.2	0.56
	R^2	0.99	0.99

Table S11.3 Fit parameters for the adsorption of Gd^{3+} ions obtained from the fit to a Langmuir and Langmuir-Freundlich type adsorption isotherm.

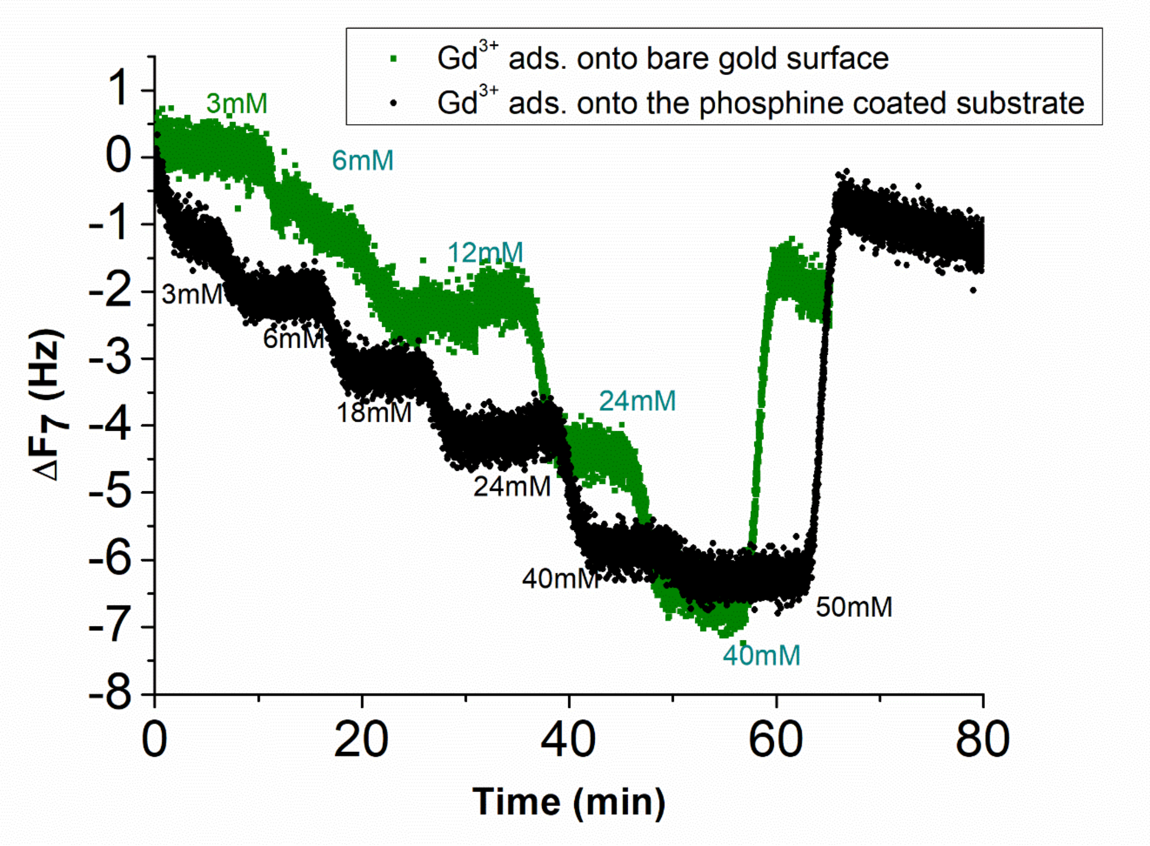

Fig. S11.4 QCM-D measurements showing the shift in frequency with time for the adsorption of Gd^{3+} ions onto a bare gold surface (green curve) and onto a BSPP precoated surface (black curve), respectively. The measurements were carried out as sequential deposition of increasing concentration of Gd^{3+} ions.

The injection onto the citrate-coated substrate of a water solution of Gd^{3+} ions (3 mM), instead of a citrate solution (24 mM) of Gd^{3+} ions (3 mM) causes the increasing of the frequency confirming the weak interaction of the citrate with the gold surface and the failure of the Gd^{3+} ions to reach the gold surface at this concentration. In comparison, the same experiment on phosphine-coated substrate causes a decrease of the frequency in agreement with the adsorption of the Gd^{3+} ions. (Fig. S11.5)

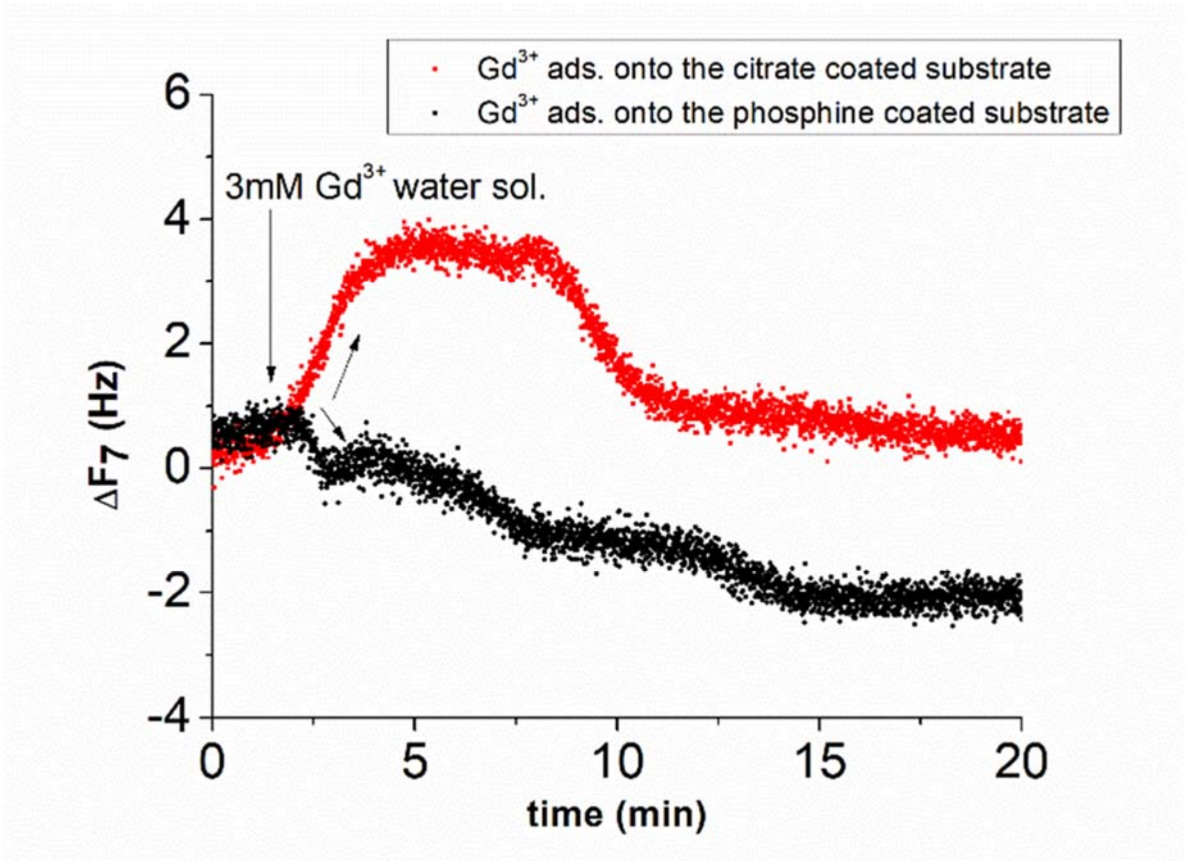

Fig. S11.5 QCM-D measurements showing the shift in frequency with time for the adsorption of 3mM aqueous solution of Gd^{3+} ions onto a citrate (red curve) and onto a BSPP precoated gold QCM-D surface, respectively.

12. TEM images of AuNP@BSPP and AuNP@citrate after incubation with Gd^{3+} ions

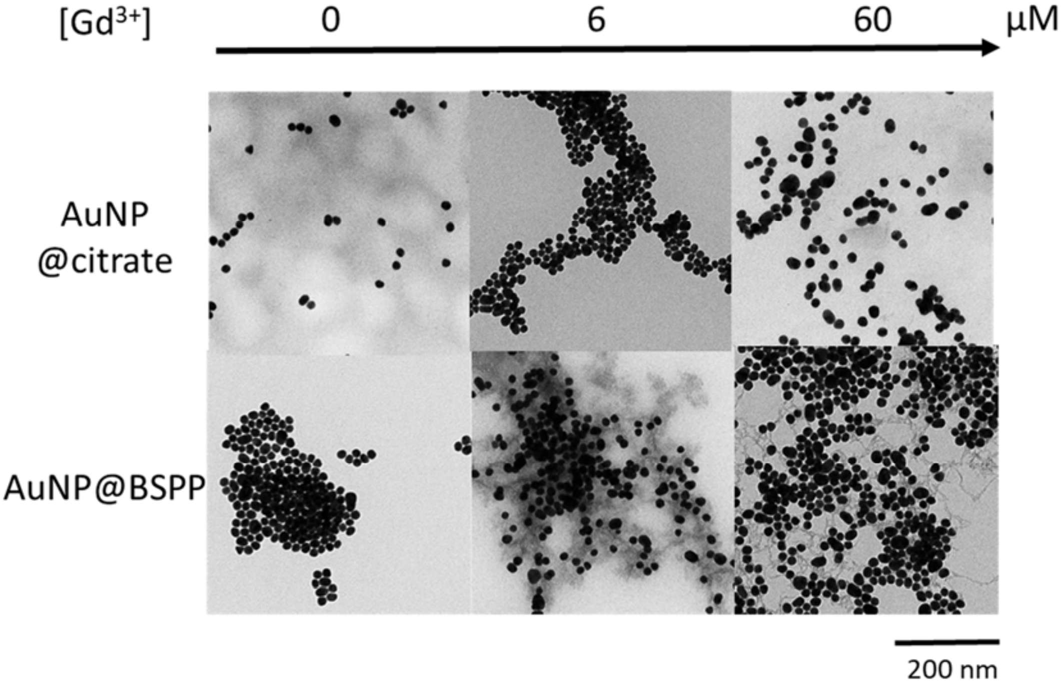

Fig S12.1 TEM images of AuNP@citrate (top) and AuNP@BSPP (bottom) before and after incubation with 6 and 60 µM of $Gd(NO_3)_3$, respectively.

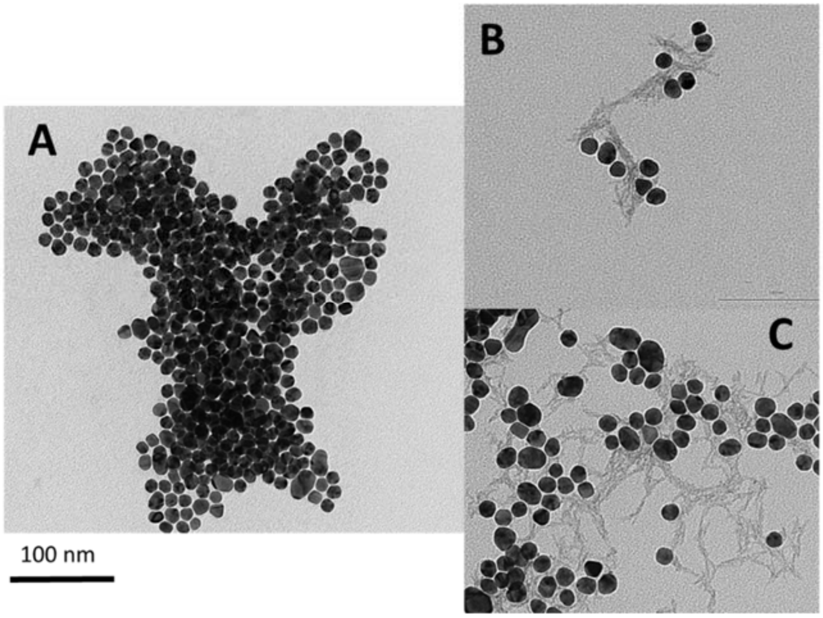

Fig. S12.2 TEM images of (A) AuNP@BSPP without adding Gd^{3+} ions, (B) AuNP@BSPP with 6 µM of Gd^{3+} and (C) AuNP@BSPP with 60 µM of Gd^{3+}.

13.Evidence of the reversibility of the aggregation of AuNP@BSPP by Gd^{3+} ions

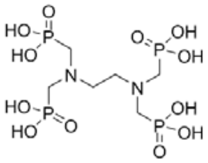

EDTMP

$[Gd^{3+}]$ =20 µM

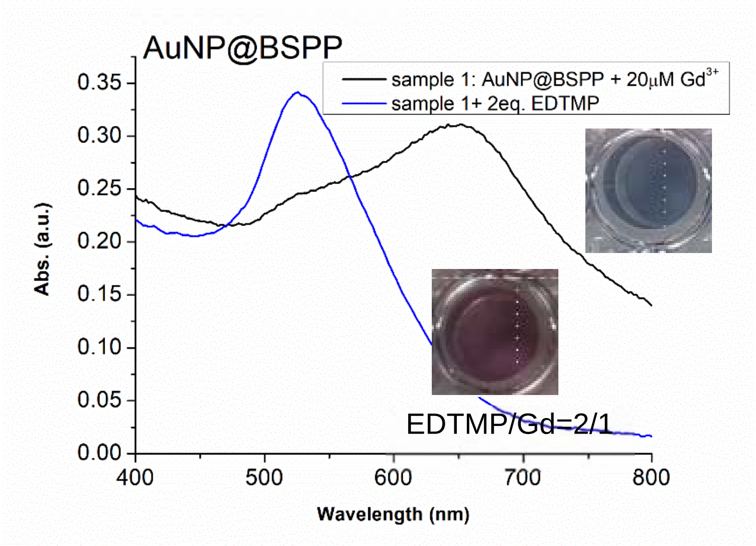

Fig. S13 UV-visible spectra of AuNP@BSPP containing 20 µM of Gd^{3+} (black curve) and of the same sample after addition of 2 equivalents of EDTMP compared to Gd^{3+} concentration (blue curve)

14. Synthesis of Hybrid Polyion Complexes (HPICs)

Copolymer solution of pEO_{5k}-b-$pAA_{3.2k}$ of $0.4\%_{wt}$ was prepared by dissolving 49mg of copolymer in 12g of Mili-Q water via ultrasonic bath. Gadolinium salt $Gd(NO_3)_3.6H_2O$ solution was prepared by dissolving 58 mg of the gadolinium salt in 11.6 g of Mili-Q water via ultrasonic bath. Five solutions of HPICs were prepared by mixing appropriate volumes of copolymer solution, gadolinium salt solution and water to reach expected ratios of positive charges over negative charges of 0.9, 1.1, 1.5, 2 and 2.5.

Filtration of HPICs solutions

Solutions of HPICs were placed into a centrifugal tube Pierce Protein Concentrator PES 3K MWCO to be centrifugated during 90 minutes at 3,000xg and 21°C. Filtrates were kept and measured for pH values. All of them were at pH around 6.

15.UV-visible spectra of AuNP@BSPP in the presence of the filtrate resulted from the purification of HPICs

Different charge ratios R (positive charges over negative charges) were used. The presence of free Gd^{3+} non-incorporated into the HPICs was detected as expected from R=1.5.

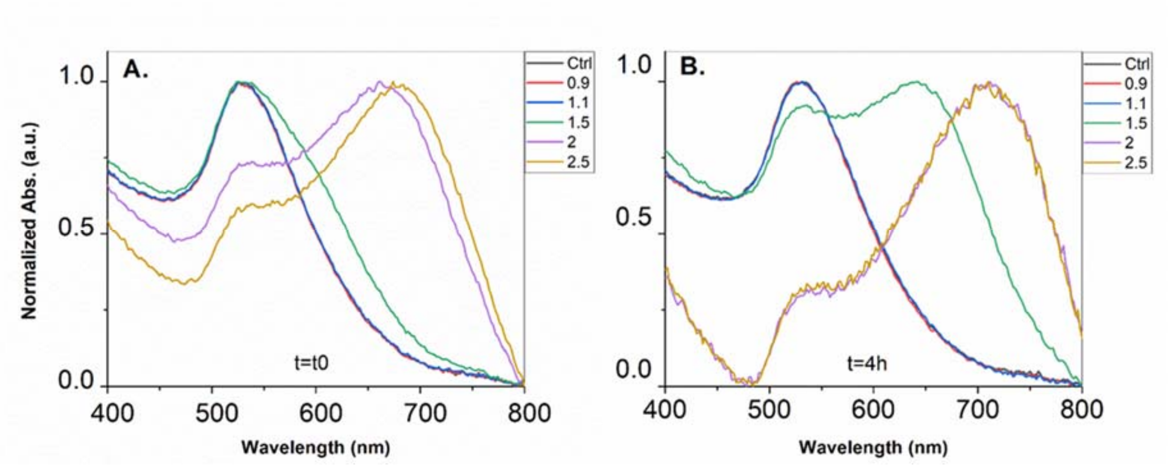

Fig. S15 UV-visible spectra of AuNP@BSPP in the presence of the filtrate resulted from the purification of HPICs of different ratios R (positive charges over negative charges) registered at t0=15min (A) and at t=4h (B).

16. Determination of the free Gd^{3+} ions concentration through the ratio of absorbance A_{640}/A_{524} as proposed in the literature .[5,6]

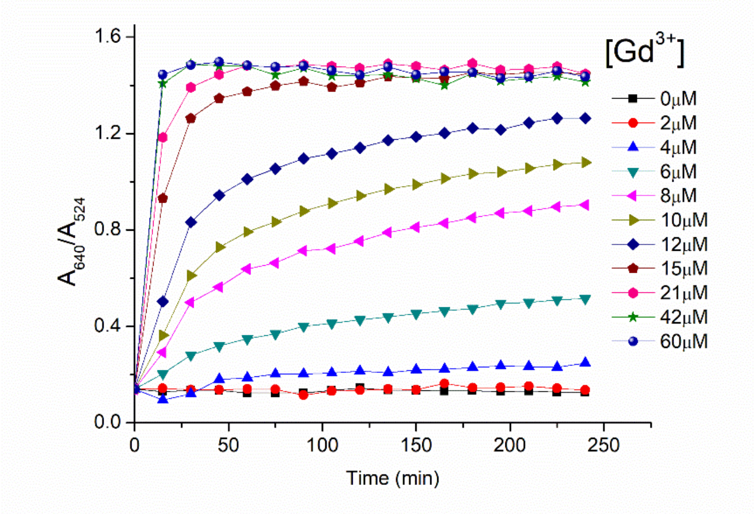

Fig. S16 .1 Time dependence of the absorbance ratio A_{640}/A_{524}

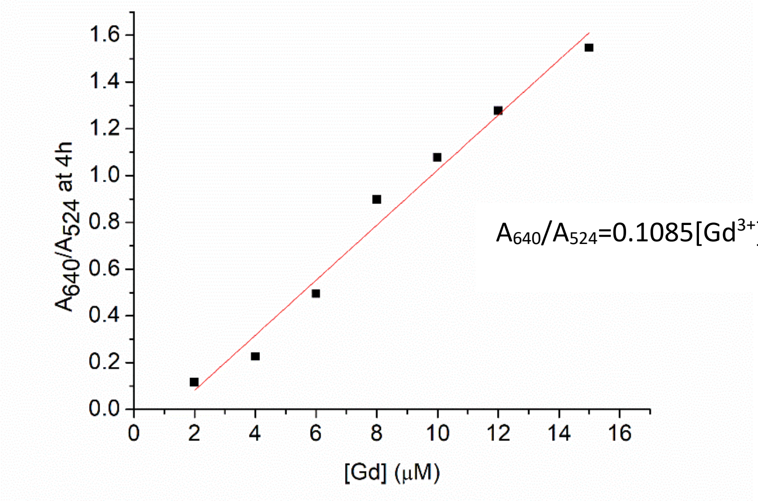

$A_{640}/A_{524}=0.1085[Gd^{3+}]-0.087$ (eq. 3)

Fig. S16.2 Linear range of the absorbance ratio (A_{640}/A_{524}) at 4h plotted vs. concentration of $Gd(NO_3)_3$.

R=3.[Gd^{3+}]/[AA] (HPICs)	Estimated concentration of free Gd^{3+} ions with eq. 3 (µM)
0.9	5
1.1	5.7
1.5	10.58
2	10.90
2.5	10.99

Table S16.3 Estimation of the concentration of free Gd^{3+} ions with the equation $A_{640}/A_{524}=0.1085[Gd^{3+}]-0.087$. The values obtained are not in agreement with the expected values.

1 W. Haiss, N.T. Thanh, J. Aveyard and D. G. Ferning, *Anal. Chem.*, 2007, **79**, 4215-4221.

2 C. Contado and R. Argazzi, *J. Chromatogr. A*, 2009, **1216**, 9088–9098.

3 I. Langmuir, *J. Am. Chem. Soc.*, 1918, **40**, 1361–1403.

4 R. Sips, *The Journal of Chemical Physics*, 1948, **16**, 490–495.

5 P. Mondal and J. L. Yarger, *The Journal of Physical Chemistry C*, 2019, **123**, 20459–20467.

6 D. Maiolo, L. Paolini, G. Di Noto, A. Zendrini, D. Berti, P. Bergese and D. Ricotta, *Anal. Chem.*, 2015, **87**, 4168–4176.
